# RBS and Promoter Strengths Determine the Cell-Growth-Dependent Protein Mass Fractions and Their Optimal Synthesis Rates

**DOI:** 10.1021/acssynbio.1c00131

**Published:** 2021-11-12

**Authors:** Fernando N. Santos-Navarro, Alejandro Vignoni, Yadira Boada, Jesús Picó

**Affiliations:** Synthetic Biology and Biosystems Control Lab, Institut d’Automàtica i Informàtica Industrial, Universitat Politècnica de València, Camí de Vera S/N, 46022 Valencia, Spain

**Keywords:** gene expression, burden, resources allocation, growth rate, RBS strength, promoter strength, protein synthesis mass fractions

## Abstract

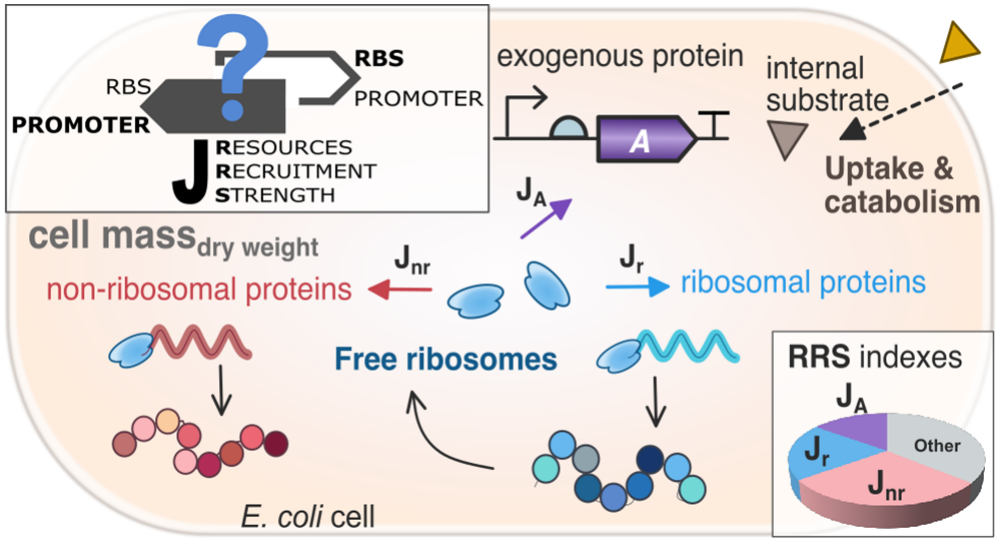

Models of gene expression considering host–circuit interactions are relevant for understanding both the strategies and associated trade-offs that cell endogenous genes have evolved and for the efficient design of heterologous protein expression systems and synthetic genetic circuits. Here, we consider a small-size model of gene expression dynamics in bacterial cells accounting for host–circuit interactions due to limited cellular resources. We define the cellular resources recruitment strength as a key functional coefficient that explains the distribution of resources among the host and the genes of interest and the relationship between the usage of resources and cell growth. This functional coefficient explicitly takes into account lab-accessible gene expression characteristics, such as promoter and ribosome binding site (RBS) strengths, capturing their interplay with the growth-dependent flux of available free cell resources. Despite its simplicity, the model captures the differential role of promoter and RBS strengths in the distribution of protein mass fractions as a function of growth rate and the optimal protein synthesis rate with remarkable fit to the experimental data from the literature for *Escherichia coli*. This allows us to explain why endogenous genes have evolved different strategies in the expression space and also makes the model suitable for model-based design of exogenous synthetic gene expression systems with desired characteristics.

## 1 Introduction

The interrelations among the cell environment from which the cell uptakes substrates, its metabolism, and the engagement of cell resources needed for gene expression result in host–circuit interactions between gene circuits and their cell host. These interactions induce competition for common shared cell resources affecting gene expression and cell growth. Endogenous genes have evolved different strategies to deal with the problem of optimal protein expression under different needs and cell conditions.^1^ As for exogenous genes, one of the fundamental problems in the rational design of synthetic genetic circuits of increasing complexity, partly explaining the current disparity between the ability to design biological systems their actual experimental performance, is the lack of systematic design methods considering the host–circuit interaction.^2^ Cells have reached an optimal use of their resources during evolution. The overexpression of exogenous genes by a genetically modified microorganism as well as the production of metabolites by the addition and/or modification of their metabolic pathways introduce a metabolic load that takes the microorganism off its natural state.^3^ The resulting competition for common shared cell resources affects cell growth and introduces spurious dynamics,^4^ leading to problems of malfunctioning of the synthetic circuit. It also triggers its elimination by evolutionary mechanisms trying to restore the natural optimal state.^5^

Mathematical models of gene expression accounting for cellular resources competition can be used to decipher the mechanisms underneath gene expression strategies that have evolved to optimize different criteria. This is not only useful to understand natural systems but also addresses the rational design of synthetic genetic circuits. Therefore, in the last years, there has been an increasing interest in the development of models and methods for model-based design of synthetic gene circuits considering host–circuit interactions.^6^

The simplest burden-aware models deal with the interactions among genes in a gene network and consider shared cell resources as an external source, without considering the host behavior. This approach has proved very useful to deal with the so-called retroactivity,^7^ the loading interaction among circuit modules and host originated from mass exchange. Retroactivity poses problems when predicting the behavior of a large network from that of the composing modules. It is a problem analogous to modeling the coupling between electrical circuits connected to a real energy source. Thus, the models accounting for it somewhat resemble Ohm’s law.^4,8^ As these models do not explicitly consider the host behavior, they cannot be easily used within a multiscale framework integrating synthetic circuits of interest, host, and cell environment at the macroscopic level.

Alternatively, one may develop models relating substrates uptake, cell growth rate, and availability of free resources as a function of the gene circuits demand. These range from very coarse-grain ones^9−12^ to semimechanistic ones with varied degrees of granularity.^13−15^ In this last case, the interplay between circuit, host, and environment can be directly incorporated into the circuit model of interest to capture the impact of cellular trade-offs and resource competition on the circuit function.

The construction of a large-scale mechanistic model of *Escherichia coli* has enabled us to integrate and cross-evaluate a massive, heterogeneous dataset integrating measurements reported by various groups over decades.^15^ On the other hand, medium-size detailed mechanistic models like the one developed in Weiße et al.^13^ have been used to study behavioral modulations of a gene switch^16^ or a feed-forward circuit.^17,18^ These medium- and large-scale models, though very useful, are most often overparametrized and cannot easily be integrated within a user-friendly and lightweight computational framework for model-based circuit design.

The small-size model presented here has enough granularity to provide good predictions of the host dynamics, the expression of the genes of interest, and their interactions while having a small number of parameters to be estimated. We derived the dynamics of gene expression as a function of the fraction of free ribosomes relative to available mature ones considering protein synthesis on polyribosomes. We also defined the gene resources recruitment strength (RRS) as the key functional coefficient that allowed us to explain the distribution of resources among the host and the genes of interest and the relationship between the use of resources and cell growth. An additional goal was to provide a model useful for model-based circuit design purposes. To this end, the model considers explicitly lab-accessible gene expression characteristics such as promoter and ribosome binding site (RBS) strengths. We derived the equivalence between the relative resources recruitment strength and the relative mass fraction of a given protein at steady state. From this equivalence, the protein synthesis rate can be easily evaluated using the average host dynamics at steady state. We used experimental data from the literature to estimate the average resources recruitment strength for both ribosomal and nonribosomal proteins in *E. coli*. This allowed us to evaluate how the sensitivity of the resources recruitment strength to RBS and promoter can explain the variation of the cell protein mass fractions with growth rate and the differential roles they play. These data also can show how host–circuit interaction shapes the optimal abundance rates of both endogenous and exogenous proteins in the expression space.

## 2 Results

### 2.1 Burden-Aware Model of Gene Expression Dynamics

**Figure 1:**
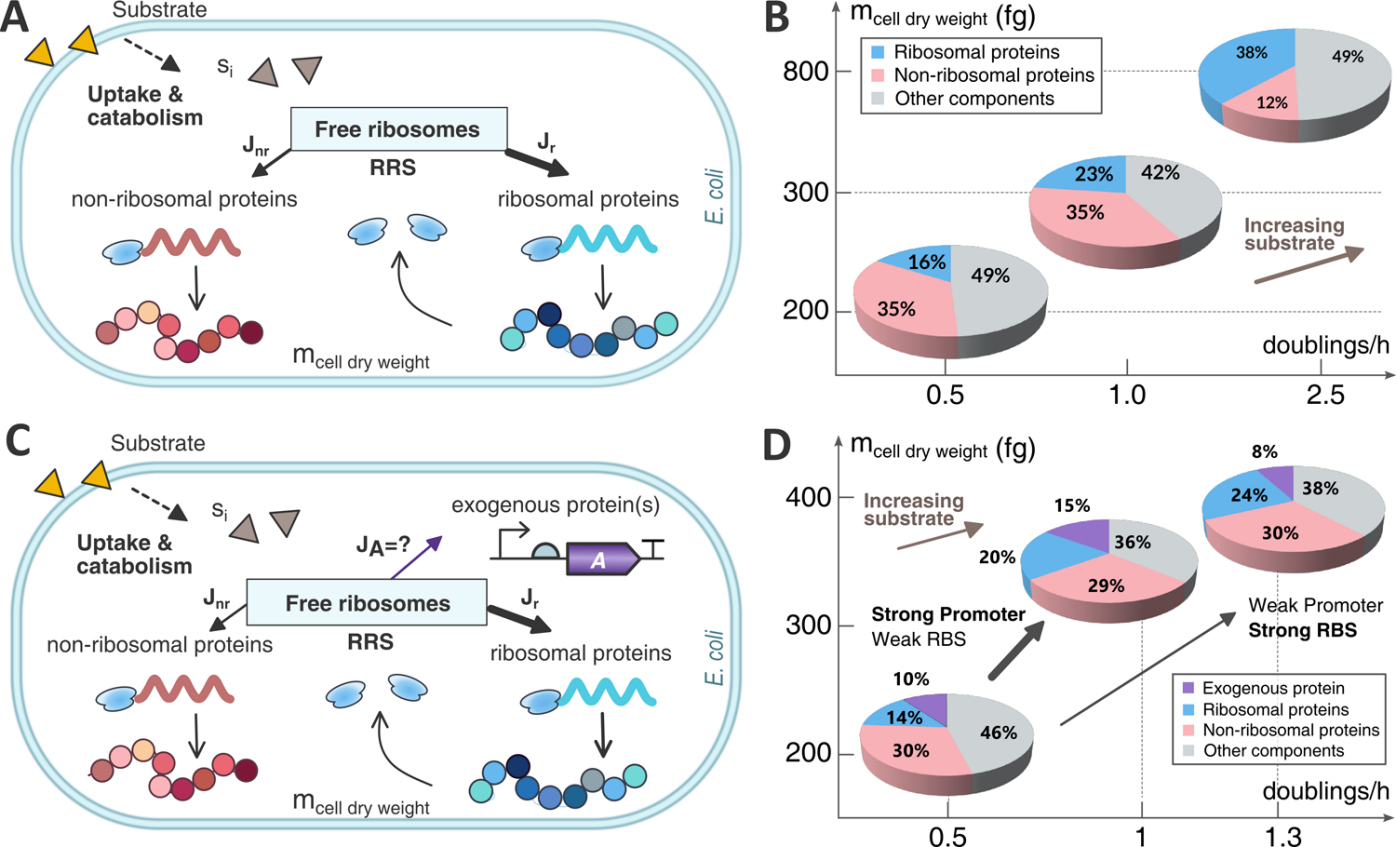
(A, C) Schematic view of the partitioned use of cell resources (ribosomes) to synthesize both ribosomal and nonribosomal proteins. The ribosomal proteins generate functional ribosome molecules to serve as the fundamental resource for protein synthesis. The resources recruitment strength (RRS) coefficient explains how the cell resources are allocated among the host endogenous genes (A) and both endogenous and exogenous genes of interest (C) (e.g., exogenous protein(s) expressed by a synthetic genetic circuit). (B) Resource allocation in a host cell in terms of the fractions of cell dry weight for the ribosomal proteins, nonribosomal ones, and other components. The pie charts represent different resources allocation scenarios, with increasing growth rates when the available substrate is increasing in the *x*-axis, and the resulting cell dry weight in the *y*-axis. (D) Resource allocation for a host cell expressing an exogenous protein. Two strategies were used for expressing the exogenous protein: strong promoter with weak RBS and weak promoter with strong RBS. The pie charts show the resource allocation distribution for both strategies (cell dry weight in the *y*-axis) for different growth rates (*x*-axis) caused when the availability of substrate is increased. Both strategies start from the same mass distribution at 0.5 doublings·h^–1^ (they share the same pie chart). The substrate was increased in the same quantity for both strategies. The arrows point to the resulting mass distribution pie chart for each strategy.

Our model considers, on the one hand, the dynamics of the expression of the cell host endogenous protein-coding genes. These are the genes that contribute to cell growth (Figure 1A). On the other hand, the model allows the possibility of considering the expression of protein-coding exogenous genes (Figure 1C). These do contribute to cell mass, but not to cell growth, akin to the consideration of unproductive proteins used in ref (9).

We started by modeling the polysomic gene expression dynamics for a generic *k*th protein-coding gene in prokaryote cells. We considered that transcription is faster than translation so it can be assumed at steady state, and that ribosomes are the limiting shared resource required for protein expression (see Section SI.1, Supporting Information (SI)). Under these assumptions, we derived the dynamics for the number of molecules of a *k*th protein as
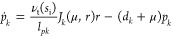
1where *p*_*k*_ is the number of copies of the *k*th protein, *l*_*pk*_ is the protein length expressed as equivalent number of amino acids, *d*_*k*_ is the protein degradation rate constant, μ is the cell specific growth rate, *r* is the number of free ribosomes, and ν_t_(*s*_i_) is the substrate-dependent effective peptide chain elongation rate. This one is expressed using the Michaelis–Menten expression
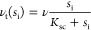
2where ν is the maximum attainable peptide synthesis rate and *K*_sc_ is a Michaelis–Menten parameter related to the cell substrate uptake and catabolic capacity. As a first approximation, we considered that ν is organism-dependent but does not depend on the sequence of nucleotides (i.e., on the particular gene being expressed) and *K*_sc_ is organism- and substrate-dependent but does not depend on the nucleotides sequence either.

The term *J*_*k*_(μ, *r*), defined as the resources recruitment strength (RRS), is a dimensionless function of the growth rate μ and the number of free mature ribosomes *r* that quantifies the capacity of a *k*th gene to engage cellular resources to get expressed (Figure 1A). It is the key functional coefficient in our model that explains the distribution of resources among the host and the genes of interest and the relationship between the usage of resources and cell growth (see its derivation in Section SI.1, SI). Besides depending on the cell growth rate and the availability of cell resources, the RRS is a function of the promoter and RBS strengths. For a generic protein-coding gene, its RRS is defined as
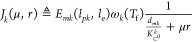
3On the one hand, the resources recruitment strength *J*_*k*_(μ, *r*) depends on the availability of cell resources: the flux of free ribosomes μ*r* and the ribosomes density-related term *E*_*mk*_(*l*_*pk*_,*l*_e_). This one is obtained (see Section SI.1, SI) as
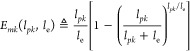
4where 1/*l*_e_ is the specific ribosomes density, with *l*_e_ expressed as equivalent number of codons. The ribosomes density can be estimated as an inversely log-linear function of the protein length *l*_*pk*_ (see eq S92 in Section SI.13, SI). Interestingly, *E*_*mk*_ can accurately be approximated as *E*_*mk*_(*l*_*pk*_,*l*_e_) ≈ 0.62*l*_*pk*_/*l*_e_ (i.e., a linear function of the number of ribosomes elongating along the transcript) for a wide range of values of *l*_*pk*_ and *l*_e_ (see Figure SI.2).

On the other hand, the RRS *J*_*k*_(μ, *r*) also depends on gene expression characteristics: mRNA transcription rate ω_*k*_(*T*_f_) or the promoter strength, mRNA degradation rate constant *d*_*mk*_ and the effective ribosome binding site (RBS) strength *K*_C^0^_^*k*^(*s*_i_). This one is defined as
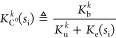
5where *K*_b_^*k*^ and *K*_u_^*k*^ are, respectively, the association and dissociation rate constants between a free ribosome and the RBS, and *K*_e_(*s*_i_) = ν_t_(*s*_i_)/*l*_e_ is the translation initiation rate constant, which depends on the availability of intracellular substrate (see Section SI.1 for details).

The resources recruitment strength of a given protein-coding gene can be related with its translation rate and number of transcripts. Consider the standard dynamic model for the expression of a protein *p*(19)
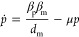
6where β_m_ (mRNA/*t*) is the transcription rate, β_p_ (protein/(mRNA·*t*)) is the translation one, and *d*_m_ is the mRNA degradation rate constant. Comparing with eq 1, we derived the relationship
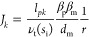
7The expression eq 7 allows us to calculate the theoretical maximum RRS, *J*_*k*_|_*r*=1_, from the available experimental data (see Section SI.12, SI).

The dynamics of the total number of ribosomes can be obtained by considering an analogous expression to eq 1 for each of the *N*_r_ proteins forming up a ribosome (see Section SI.2, SI). The total number of ribosomes in the cell at any one time instant, *r*_T_, is the sum of the mature (*r*_a_) and inmature (*r*_i_) ones. In turn, the mature ribosomes *r*_a_ available for translation comprise the free ribosomes *r* and the ones already bound either to the RBSs or elongating along the transcripts. The number of available mature ribosomes is a fraction of the total number of ribosomes so that *r*_a_ = Φ_m_*r*_T_. We assumed the fraction Φ_m_ varies little in time (see Section SI.2, SI) so that the dynamics of the total number of ribosomes and that of the number of available ribosomes are the same but for a scale factor. Without loss of generality, we considered average protein-coding endogenous genes with RRSs *J*_r_ and *J*_nr_, respectively. This allowed us to obtain the relationship between free and total number of ribosomes (see Section SI.3, SI) as
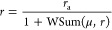
8with
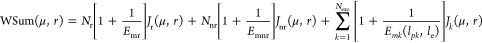
where *N*_r_ and *N*_nr_ are the number of ribosomal and nonribosomal protein-coding endogenous genes, respectively, and *N*_exo_ allows for the existence of exogenous genes.

Cell growth can essentially be explained as the time variation of the protein fraction of the total cell mass (Figure 1B). Yet, not all protein mass contributes to cell growth. There are proteins which may be undergoing active degradation while other proteins, e.g., the exogenous ones will not have any active role positively contributing to the cell growth. Therefore, we considered only the endogenous ribosomal and nonribosomal proteins to compute the cell specific growth rate. We used the dynamics eq 1 and assumed an average amino acid mass *m*_aa_ to obtain the time variation of the total endogenous protein mass content *m*_h_ of the native host cell (see Section SI.5, SI)
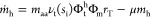
9where
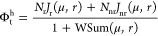
10is the fraction of ribosomes elongating along endogenous ribosomal and nonribosomal transcripts relative to the total number of mature available ribosomes (see Section SI.4, SI).

Next, we considered that the total biomass dry weight variation (i.e., that of the whole population of cells) is mainly caused by cell duplication (i.e., population growth), and the dynamics of cell mass accumulation are much faster than those of cell duplication. Under this assumption, the protein mass for each cell quickly reaches steady state (*ṁ*_h_ ≈ 0). Thus, from eq 9, we obtained the expression for the cell specific growth rate
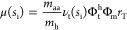
11where note that Φ_t_^h^Φ_m_*r*_T_ is the number of ribosomes actively elongating along endogenous transcripts at a given time instant (see Sections SI.4 and SI.5, SI).

To relate the growth rate μ(*s*_i_) obtained from the intracellular dynamics with the extracellular measure of growth rate, μ(*s*), derived from cell population dynamics, we followed a reasoning derived from the model developed in ref (13), where the quantity of intracellular substrate *s*_i_ is related to the one of extracellular substrate *s* through the dynamics of nutrient import and catabolism (see Section SI.6 for details). Under the condition of steady-state growth where the rate of total cell mass growth is identical to the rate of cell number growth^20^ and assuming that the maximum import and catabolism fluxes are balanced, we obtained the Monod population growth kinetics
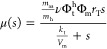
12where *V*_m_ is a parameter related to the effective volume of culture broth for each cell and *k*_t_ is the Michaelis–Menten constant for substrate transport into the cell. Note that we recuperate the maximum specific growth rate μ_m_ as a linear function of the number of ribosomes actively elongating along transcripts at a given time instant. Finally, the Monod constant *K*_s_ as a function of the substrate transport capacity and the cell harvesting volume.

Our model accounts for the protein mass distribution (Figure 1B) but does not consider the relationship between growth rate and the total cell protein mass. Cells growing at faster growth rates are larger and heavier, thus affecting their total protein content.^21^ To model the relationship between the cell protein content and the specific growth rate for the native host cell—i.e., *m*_h_ = *m*_h_(μ)—we postulated the relationship
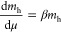
13with *m*_h_(0) = 77.375 fg and β = 61.781 min as best fits obtained for *E. coli* cells using the data in ref (22). We also considered an analogous model relating the cell dry weight *m*_h,cDW_(μ) with the growth rate (see Section SI.7 for details).

Finally, we structured our model in such a way that it can be used to analyze the resource allocation trade-offs (see Figure 1D) among the endogenous protein-coding genes from the native *E. coli* host cell, and a given set of exogenous ones of interest (e.g., a synthetic gene circuit). In the latter case, we have considered, without losing generality, a single exogenous protein of interest *A* to exemplify the model expressions and the interaction between the host cell and the exogenous additions.

For the endogenous protein-coding genes, we considered the ensemble of ribosomal and nonribosomal genes as lumped species with average values of *E*_mr_(*l*_p_^r^,*l*_e_), *E*_mnr_(*l*_p_^nr^,*l*_e_) and *J*_r_(μ, *r*), *J*_nr_(μ, *r*), respectively. Then, we obtained the dynamics of the total mass of ribosomes *m*_*r*_T__ and nonribosomal endogenous proteins *m*_nr_, and the dynamics of the mass *m*_A_ of the exogenous protein (see Sections SI.8 and SI.9 for details) as
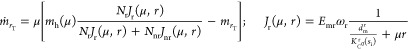
14
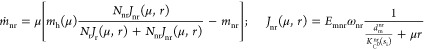
15
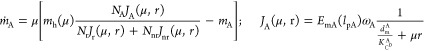
16where *N*_A_ is the gene copy number of A, the number of free ribosomes *r* is obtained using eq 8, and the specific growth rate μ is calculated using eq 11.

The denominator in the fraction of RRSs only includes the host protein-coding genes. The protein mass *m*_h_(μ) is that of the native host cell, comprising only the cell endogenous proteins. We defined the mass of the strain *m*_s_ = *m*_h_ + *m*_A_ as the one comprising the mass of the host and that of the exogenous proteins. We obtained the relation between the protein mass of the strain *m*_s_(μ) and that of the native host *m*_h_(μ) (see Section SI.9, SI) as
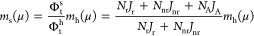
17In addition, we also considered the cell dry weight *m*_cDW_(μ), comprising the mass *m*_h_(μ) of the endogenous ribosomal and nonribosomal proteins, the mass of the exogenous proteins *m*_A_(μ) and the mass of other constituents of the cell, denoted as *m*_Q_(μ). Thus, *m*_cDW_(μ) = *m*_h_(μ) + *m*_A_(μ) + *m*_Q_(μ) = *m*_s_(μ) + *m*_Q_(μ). To obtain *m*_Q_(μ), we used the estimation of the cell dry weight *m*_h,cDW_(μ) for the *E. coli* host native cell, i.e., without expression of exogenous genes (see Section SI.7 for details), assuming that *m*_Q_(μ) does not depend on the expression of exogenous genes. This allowed us to estimate the mass fractions with respect to the total cell dry weight.

To evaluate the productivity rate of a given protein of interest, we obtained its mass synthesis rate as the steady-state mass of protein produced per cell and generation (see Section SI.9, SI). In the case of an exogenous protein *A* and using eqs 16 and 17, we obtained
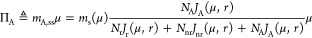
18We defined the specific mass synthesis rate (spMSR) relative to the cell dry weight as
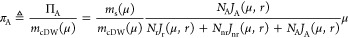
19For a given protein *A*, both the protein mass synthesis rate (g·min^–1^) and the specific one (g·min^–1^·gCDW^–1^) are directly related to its relative RRS fraction.

From the results above, we obtained the cell specific growth rate at steady-state exponential balanced growth as
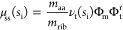
20where *m*_rib_ is the average ribosome mass (see Section SI.9 for details). That is, the cell growth rate at steady state depends linearly on the fraction Φ_t_Φ_r_^t^ of bound ribosomes being actively used to build up ribosomes themselves (i.e., ribosomes actively elongating along and translating ribosomal transcripts) relative to the total number of ribosomes.

### 2.2 Ribosomal and Nonribosomal Genes Differ in Their Average Resources Recruitment Strength

Using the experimental data in ref (19), we evaluated the maximum expected magnitude of the resources recruitment strength for each gene using eq 7 with *r* = 1, i.e., the theoretical maximum RRS for a given availability of intracellular substrate, *J*_*k*,max_ = *J*_*k*_|_*r* = 1,ν_t_(*s*_i_)_. The data in ref (19) correspond to *E. coli* cells under fast-growing conditions (doubling time *t*_d_ = 21.5 min). Therefore, we could assume saturation of substrate, allowing us to consider the maximum substrate-dependent effective translation elongation rate ν_t_(*s*_i_) = ν to evaluate eq 7. Note that this is equivalent to estimating the maximum RRS for the maximum specific growth rate. *E. coli* has around 4225 protein-coding genes.^23,24^ From ref (19), we obtained data for a representative enough set of genes, comprising 3551 nonribosomal and 68 ribosomal ones, accounting for around 86% of all *E. coli* genes.

First, the results allowed us to estimate the order of magnitude of the resources recruitment strength for ribosomal and nonribosomal genes in *E. coli* and their maximum average value. Then, we obtain how many genes of each class are active at any one time.

**Figure 2:**
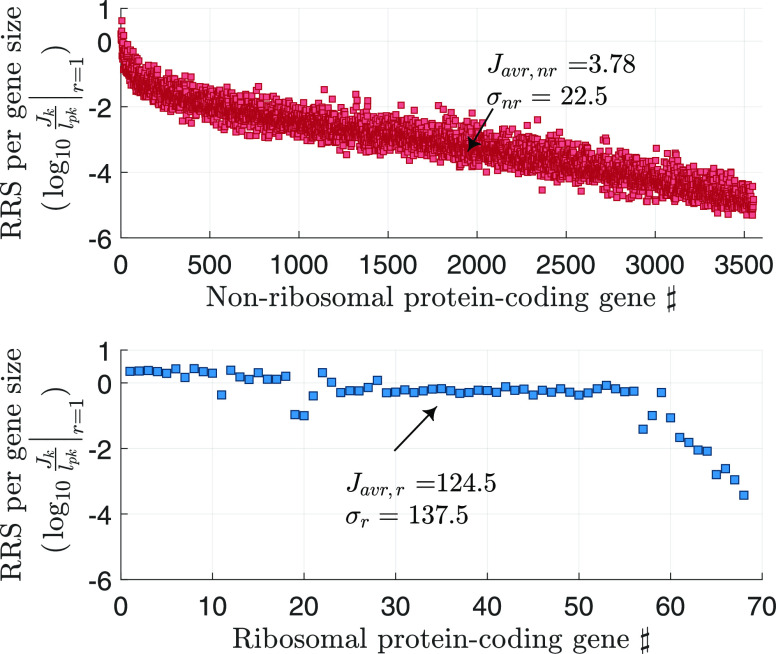
Log-magnitude of the ratio between the maximum resources recruitment strength and the length (aa) of the associated protein for the set of nonribosomal (top) and ribosomal (bottom) protein-coding genes in ref (19). The genes were ordered by decreasing value of the ratio.

As expected, the values obtained spanned several orders of magnitude. For the ribosomal genes, the average value *J*_max,r_^avg^ = 124.5 and a coefficient of variation CV_*J*_max,r__ ≈ 1, while for the nonribosomal ones, the values were *J*_max,nr_^avg^ = 3.78 and CV_*J*_max,nr__ ≈ 6. The average maximum RRS for the ribosomal genes was 2 orders of magnitude higher than for nonribosomal ones. Yet, the coefficient of variation was much smaller for the ribosomal resources recruitment strengths than for the nonribosomal ones. Figure 2 shows the values of *J*_*k*,max_ we obtained for each gene sorted by the log-magnitude of the ratio between the maximum RRS and the length of the associated protein. The results did not essentially change from the non-normalized ones (see Figure SI.6, SI). That is, the resources recruitment strength of *E. coli* genes is not fundamentally determined by the lengths of the proteins they code. This suggests that factors such as the effective transcription and translation rates are more relevant.

But not all genes are expressed all of the time. As a proxy to estimate how many genes are active at any given time, we calculated the cumulative sum of the maximum RRS and obtained how many genes being expressed are required to explain both 95 and 99% of the total cumulative sum (see Figure SI.8). We did this independently for both ribosomal and nonribosomal proteins. Our results showed that out of the 68 ribosomal genes, 49 of them (72%) explained 95% of the cumulative sum of the maximum resources recruitment strength. To explain 99%, we needed 57 ribosomal genes (84% of them). However, for nonribosomal genes, we needed 875 out of 3551 genes (25%) to explain 95% of the cumulative sum and 1735 (49%) to explain the 99%.

### 2.3 Resources Recruitment Strength Explains the Distribution of Ribosomal and Nonribosomal Protein Mass Fractions

The relative mass fractions of ribosomal and nonribosomal proteins in the cell depend on the cell growth rate so that the ribosome content increases linearly with growth rate.^9,22,25,26^ Existing resource allocation models explain this as a result of optimal allocation of cell resources between the ribosomal and nonribosomal fractions, balancing the demands of protein synthesis and nutrient influx under the constraint that the sum of both fractions remains constant.^9^ In our model, the relative resources recruitment strength of a given protein equals its relative mass fraction in the cell at steady-state balanced growth (see eqs 14–16 and Section S.8, SI). Therefore, the relative distribution of mass between ribosomal and nonribosomal proteins must be reflected in the relative distribution of their resources recruitment strengths.

We first studied the *E. coli* host cell, i.e., without any exogenous protein-coding genes. We used the data in ref (22) to check whether our model was able to predict the linear increase of ribosomes content with growth rate and the relative distribution of endogenous ribosomal and nonribosomal protein mass fractions as a function of growth rate. We did not estimate the model parameters to try and directly fit the experimental relative distribution of resources recruitment strengths, as this would not inform on the capability of the model to capture the intrinsic relationship among growth rate, use of cell resources, and distribution of protein mass fractions. Instead, we analyzed if a good fit of the specific growth rate implied our model could generalize and predict the relative mass fractions in the cell. This, in turn, implies fitting the ribosomal and nonribosomal resources recruitment strengths.

To this end, we fitted the model parameters using the experimental growth rate as output to predict. We used the values of the peptide chain elongation rates ν_t_(*s*_i_) as a function of growth rate available from ref (22) as the only input information given to the model. This is tantamount to feed the model only with the available amount of substrate *s*_i_ (see Section 5.2). Then, we minimized the sum over the experimental data points of the absolute growth rate prediction error (see Section 5.2). We considered the lumped resources recruitment strengths for both the ribosomal and nonribosomal endogenous proteins (see expressions 14 and 15) and estimated the fraction of mature ribosomes and the parameters corresponding to the RBS strength and transcription rates. This would provide our model a good fit of the specific growth rate. The best-fit estimated parameters are given in Table 1.

**Table 1:** Average Best-Fit Estimated Values for *E. coli* of the RBS-Strength-Related Parameters *K*_b_^*k*^, *K*_u_^*k*^ and Transcription Rates ω*_k_* for Ribosomal (*k* = r) and Nonribosomal (*k* = nr) Proteins and the Fraction Φ_t_ of Mature Ribosomes with respect to the Total Number of Ribosomes

parameter (units)	*K*_u_^r^ (min^–1^)	*K*_u_^nr^ (min^–1^)	*K*_b_^r^ (molecule^–1^·min^–1^)	*K*_b_^nr^ (molecule^–1^·min^–1^)	ω_r_ (mRNA·min^–1^)	ω_nr_ (mRNA·min^–1^)	Φ_m_ (adim.)
mean	129.9	3.09	5.57	12.86	5.65	0.028	0.90
std.	4.07	0.14	0.78	1.50	0.29	0.25 × 10^–3^	0.5 × 10^–2^

The estimated values of the RBS-strength-related parameters *K*_b_^*k*^, *K*_u_^*k*^ implied ribosomal RBSs much weaker than the nonribosomal ones. Interestingly, the values we obtained for the transcription rates were in the same order of magnitude as the mean values obtained from the data in ref (19)—ω_r_ = 2.4 and ω_nr_ = 0.05, respectively. Therefore, this demonstrates a much higher value for the average transcription rate of ribosomal proteins than for the nonribosomal ones. Our results also estimated an average high transcription–low translation rate expression strategy for the ribosomal endogenous genes and the opposite strategy for the nonribosomal ones.

**Figure 3:**
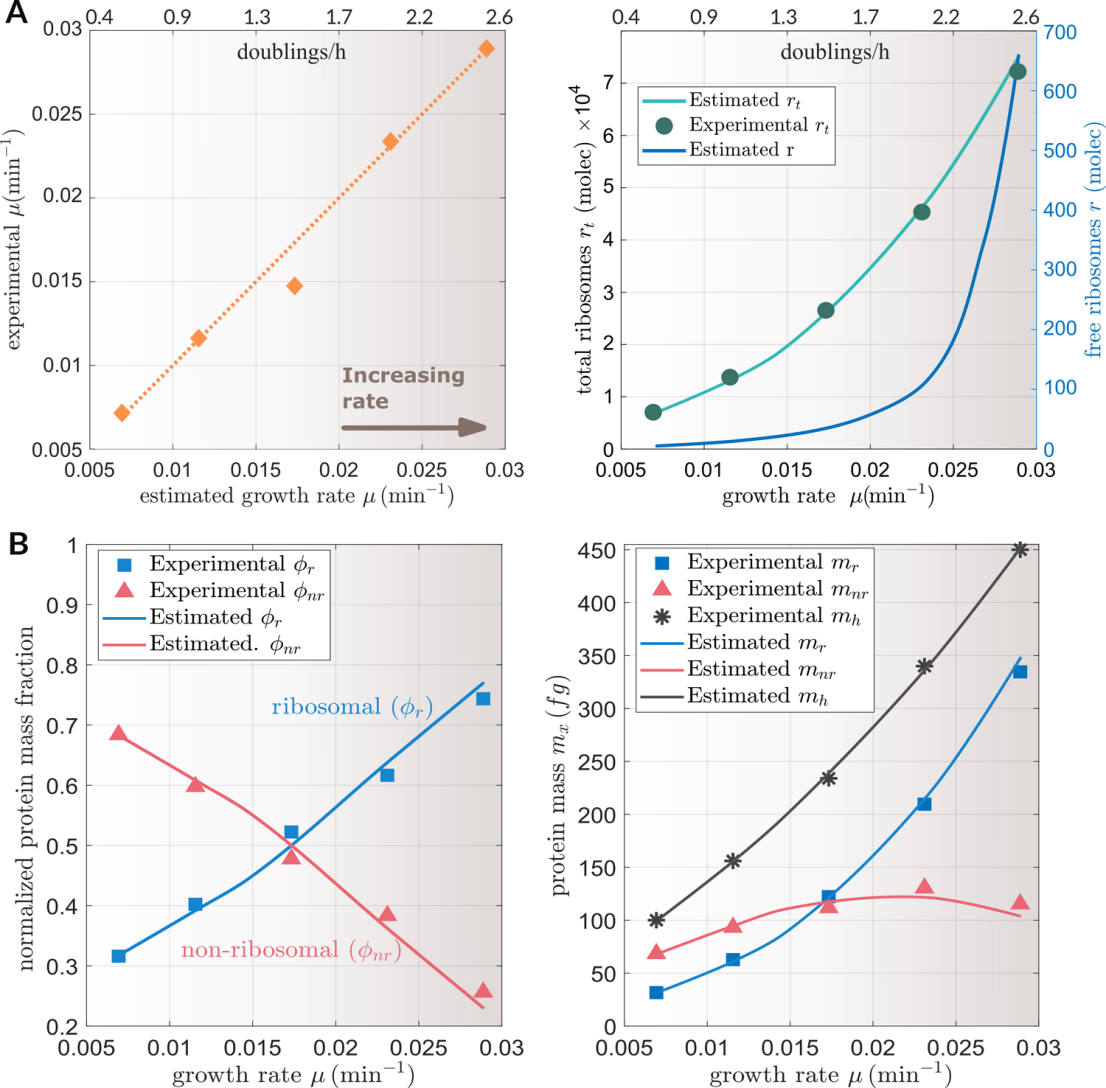
(A) Estimated versus experimental growth rate (left). Experimental and estimated number of total ribosomes as a function of the growth rate and estimated number of free ribosomes (right). (B) Estimated versus experimental mass fractions of ribosomal (ϕ_r_ = *m*_r_/*m*_h_) and nonribosomal (ϕ_nr_ = *m*_nr_/*m*_h_) proteins in *E. coli* (left). Ribosomal (*m*_r_), nonribosomal (*m*_nr_), and total host cell protein mass (right). In all plots, the *x*-axis corresponds to the estimated and experimental growth rates evaluated for the range of peptide chain elongation rates ν_t_(*s*_i_) extracted from ref (22).

Figure 3A shows the results of the model parameter fitting and the good agreement between the experimental and the estimated growth rate. The estimation of the number of free ribosomes for cells growing at doubling time *t*_d_ = 25 min (μ ≈ 0.028 min^–1^) was consistent with the result *r* ≈ 350 obtained using the experimental data in ref (19) (see Section SI.14, SI). For cells growing faster, the number of free ribosomes much increased. Note, though, that also the total number of ribosomes (both experimental and estimated) greatly increased for very fast-growing cells. Thus, the fraction of free ribosomes with respect to the total number only increased from 0.08 up to 1.37% for cell doubling times between 100 and 24 min, respectively (even though the number of free ribosomes varied by almost 200-fold). Similarly, the computed fraction Φ_m_ of mature ribosomes with respect to the total number of ribosomes was consistent with the estimated fraction of active bound ribosomes Φ_t_^h^Φ_m_ ≈ 0.78 (see Figure SI.11) in agreement with refs (12) and (22).

We evaluated the mass fractions of the endogenous ribosomal and nonribosomal proteins at steady state using the expressions 14 and 15. The model predictions were in very good agreement with the experimental values, as shown in Figure 3B. Therefore, our model reproduced the known linear increase of the ribosomal fraction with growth rate. The differential behavior between the ribosomal and nonribosomal resources recruitment strengths was behind the differential protein mass distribution as the cell growth rate increases.

**Figure 4:**
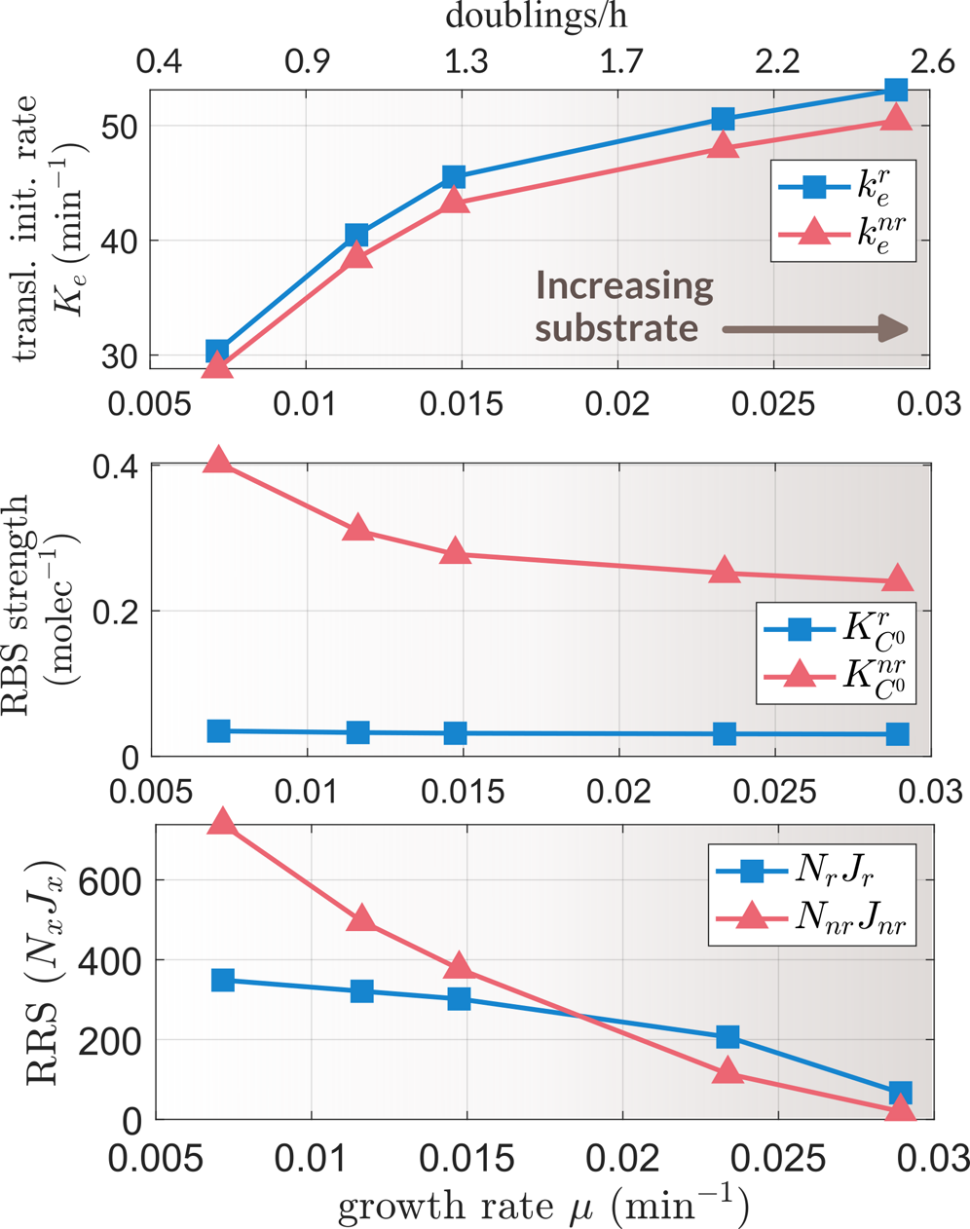
Estimated translation initiation rate *k*_e_ for the average ribosomal and nonribosomal endogenous genes as a function of the specific growth rate μ (top). Estimated effective RBS strengths *K*_C^0^_^r^ and *K*_C^0^_^nr^ (middle). Estimated total resources recruitment strengths *N*_r_*J*_r_ and *N*_nr_*J*_nr_ as a function of growth rate μ (bottom).

The effective RBS strength used in our model is a function of the intracellular substrate because it varies with the cell growth rate according to eq 5. Figure 4 shows the estimated values as a function of the specific growth rate μ. The estimated effective RBS strength of the nonribosomal protein-coding genes (*K*_C^0^_^nr^) was much higher than that of the ribosomal ones (*K*_C^0^_^r^). As the growth rate increased—tantamount in our model to an increasing intracellular substrate *s*_i_—the ribosomal effective RBS strength kept almost constant (with a slight decrease around 12%) while the nonribosomal one decreased by almost a 40%. We could explain this trend as a result of the difference in the ratio between the transcript degradation rate and the RBS strength, *d_mk_*/*K*_C^0^_^*k*^ for both ribosomal and nonribosomal genes. The ribosomal genes kept much higher values of *d*_*mk*_/*K*_C^0^_^*k*^ for all values of the flux of free resources μ*r*. This, taking into account the monotonous increasing power-law relationship between the growth rate and the number of free ribosomes predicted by our model (see Section SI.14, SI) implies the observed trends in the values of the RRS in Figure 4 (bottom). The ribosomal RRS *J*_r_(μ,*r*) decreases much slower than that of the nonribosomal ones as the growth rate increases.

Section 2.1 shows that in endogenous genes, steady state is reached for balanced exponential growth when their relative fraction of resources recruitment strength equals their mass relative to that of the host cell. Since the ribosomal resources recruitment strength decreases much slower than the nonribosomal one as the growth rate increases, the fraction of ribosomal RRS with respect to the total sum of ribosomal and nonribosomal RRSs will increase. As a consequence, its relative mass fraction will increase.

It is important to stress again that we estimated the parameters in our model so as to fit not the experimental mass fractions but the cell growth rate. By doing that, the internal structure of the model—substantiated in the structure of the resources recruitment strength functional coefficients—captured the correct differential mass distribution between ribosomal and nonribosomal cell protein content as a function of growth rate.

### 2.4 Host–Circuit Interaction Shapes the Optimal Synthesis Rate of Exogenous Proteins

There are multiple ways to increase the expression of an exogenous protein of interest, including the choice of the expression vector of the synthetic gene circuit, optimizing the use of codons, co-expression of chaperones to aid protein folding, etc.^27^ We focused on varying the expression space—i.e., the gene induction space defined by the values of the mature mRNA synthesis rate and the effective RBS strength—at the same values of cell growth conditions and intracellular substrate availability. We used the average host dynamics at steady-state balanced growth to evaluate the distribution of cell mass fractions and the specific protein mass synthesis rate (specific synthesis rate for short or spMSR) of a given exogenous protein of interest *A* as defined in eq 19 (see also Section SI.9) as a function of variations in the expression space.

We first considered the RBS-strength-related parameters *K*_b_^A^, *K*_u_^A^ of the exogenous gene to be constant with values equal to the estimated averages for an endogenous nonribosomal protein in *E. coli* (see Table 1) and only the mRNA synthesis rate was varied. To this end, we changed the gene copy number times the transcription rate (or promoter strength) *N*_A_ω_A_ in the range [10^–1^, 10^5^] times the average promoter strength of endogenous nonribosomal genes given in Table 1. This gave a maximum value *N*_A_ω_A_ ≈ 3.3 × 10^3^ mRNA·min^–1^, which is an attainable value for *E. coli* considering an average transcription rate ω_A_ = 3 mRNA·min^–1^ and a high copy number plasmid with *N*_A_ = 1100.

**Figure 5:**
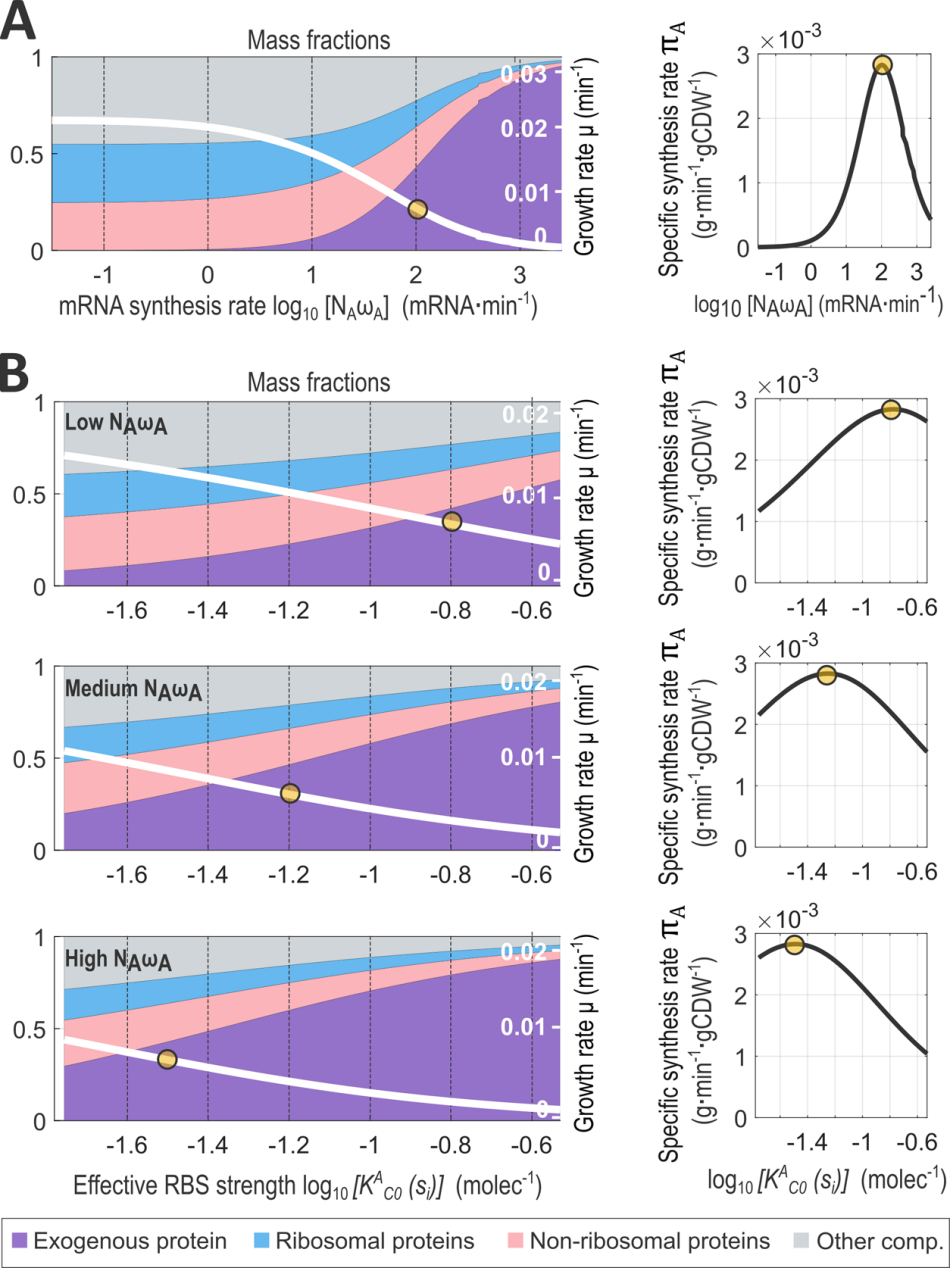
(A) Left: effect of increasing the mRNA synthesis rate of an exogenous protein A on the cell growth rate (right axis) and on the cell mass fractions (left axis). Right: specific protein mass synthesis rate (spMSR) for the exogenous protein A as a function of its mRNA synthesis rate. Even though the growth rate decreases for increasing mRNA synthesis rates, the spMSR increases, reaching a maximum value (yellow dot) at fast mRNA synthesis rates around 2000 mRNA·min^–1^ and eventually decreases for larger mRNA synthesis values. (B) Left: Effect of varying the RBS strength on the cell growth rate (right axis) and the protein mass fractions (left axis) for three increasing values of the mRNA synthesis rate (low, medium, high). Right*:* spMSR of the exogenous protein as a function of RBS strength variation.

Figure 5A shows the variation across the mRNA synthesis space *N*_A_ω_A_ of the mass fractions and the cell growth rate (left) and the spMSR, π_A_, of the exogenous protein (right). The distribution of mass fractions was consistent with the behavior of the cell. As the mRNA synthesis rate of the gene A was increased (moving toward the right in the plot Figure 5A, left), the mass fraction corresponding to the protein A also increased (purple) while that of ribosomal proteins decreased (blue) with a corresponding decrease in the cell growth rate (white line). Consequently, there appeared a maximum specific protein mass synthesis rate value (Figure 5A yellow dot, π_A_ ≈ 2.8 × 10^3^ g·min^–1^·gCDW^–1^) which was achieved for an mRNA synthesis rate of 100 mRNA·min^–1^. This value represents a low copy plasmid number *N*_A_ ≈ 20 and a constitutive promoter with a transcription rate ω_A_ ≈ 5 mRNA·min^–1^.

The model predicted an increasing mass fraction of the protein A as we continue increasing the value of the mRNA synthesis rate. However, this situation happens at the cost of reducing the fraction of ribosomal proteins, resulting in a very small growth rate. The relationship between the fraction of exogenous protein and growth rate in our model is a decreasing exponential (something consistent given its mathematical smooth differential continuous-time nature). Therefore, even if the zero growth rate is achieved in the limit for 100% of exogenous protein, note that this is a theoretical point only achieved in the limit, i.e., at infinite cell doubling time. In practice, the cell viability will be lost before.

Figure 5B (left) shows the results obtained when we analyzed three representative values of the mRNA synthesis rate *N*_A_ω_A_ = {150, 400, 800} corresponding to an average transcription rate in *E. coli* (ω_A_ ≈ 3 mRNA·min^–1^) combined with a low, medium, and high plasmid copy number, respectively. Then, we varied *K*_b_^A^, *K*_u_^A^ in the ranges considered in Section 5.2 to obtain a range of values for the effective RBS strength *K*_C^0^_^A^(*s*_i_). The mass fraction corresponding to protein A increased and the cell growth rate decreased for high levels of the RBS strength. Figure 5B (right) shows that the main factor affecting the spMSR is the mRNA synthesis rate *N*_A_ω_A_. Thus, for low values of the mRNA synthesis rate, the spMSR increased for strong RBSs until a maximum appeared for one of the stronger ones (e.g., *K*_C^0^_^A^ = 10^–0.8^ = 0.15 molecule^–1^). For medium values of *N*_A_ω_A_, there soon appeared a maximum spMSR for the exogenous protein as a function of the RBS strength. Finally, for high values of the mRNA synthesis rate, increasing the RBS strength rapidly produced a decrease in the specific protein mass synthesis rate. Our model correctly predicted that there is a critical (optimal) protein synthesis rate that is achieved for lower RBS strength as the mRNA synthesis rate increases.

The location of the optimal spMSR as a function of variations in the full range of the expression space can be seen in Figure S.12C in Section SI.15, which shows the variation of the specific synthesis rate of the exogenous protein across the expression space *N*_A_ω_A_, *K*_C^0^_^A^(*s*_i_) in log–log scale. The optimal subspace corresponded to a line in the log–log promoter–RBS space, showing the existence of a trade-off between the mRNA synthesis rate (tantamount to the gene induction) and the RBS strength. The pronounced slope of the optimal subspace explains the different sensitivity of the specific synthesis rate to the variations of either the promoter or the RBS strengths that were obtained in Figure 5A,B. Our model predicted that the specific synthesis rate is more sensitive to variations of the mRNA synthesis rate than to variations of the RBS strength. Thus, for intermediate values of *N*_A_ω_A_, there is a wide range of RBS strengths that keep the specific synthesis rate close to its optimal value. This is also reflected in the smoother transition between the mass fractions resulting when the RBS strength is modified compared to changing the mRNA synthesis rate. Note that, as predicted by eq 19, for a given substrate availability, different expression strategies resulting in the same specific synthesis rate will correspond to the same distribution of mass fractions.

Differently from modifying the mRNA synthesis rate for a fixed RBS strength value, or vice versa, the maximum spMSR of the exogenous protein significantly changed when the substrate availability does not remain constant. The effect of the differential role of RBS and promoter combinations for scenarios with varying substrate is analyzed in the next section.

### 2.5 Substrate Level Emphasizes the Differential Role of RBS and Promoter Strengths

It is well known that varying combinations of transcription and translation rates affect the stability of metabolic networks^28^ and the trade-off between desired expression levels and noise^19^ and between expression of endogenous and synthetic genes and growth.^11,13^ In the previous section, we showed that for a constant availability of substrate rich in nutrients, there are different promoter and RBS combinations that can achieve the same expression level (tantamount the same specific protein mass synthesis rate) of an exogenous protein A. This leads to a multimodal design problem. One can choose between design strategies ranging from using a combination of a weak promoter strength and a strong RBS (*N*_A_ω_A_ ↓ *K*_C^0^_^A^ ↑) to using a strong promoter and a weak RBS (*N*_A_ω_A_ ↑ *K*_C^0^_^A^ ↓). The results depicted in Figure 5 show that for the case of constant substrate, there is no difference between using one promoter–RBS combination or another as long as the desired spMSR of the protein A remains the same.

However, changes in the substrate have a different impact on the protein expression depending on which one is the promoter–RBS combination selected. Figure 1B,D illustrates how the mass fractions of ribosomal, nonribosomal, and exogenous proteins change as a function of the growth rate μ, which is indirectly dependent on the availability and quality of the substrate. For a given gene following the weak-RBS strong-promoter strategy (the one followed by the endogenous ribosomal genes), the mass fraction corresponding to the exogenous protein increased as the availability of substrate increased. On the contrary, the strong-RBS weak-promoter strategy (as followed by the endogenous nonribosomal genes) caused the exogenous protein mass fraction to decrease with increasing availability of substrate.

**Figure 6:**
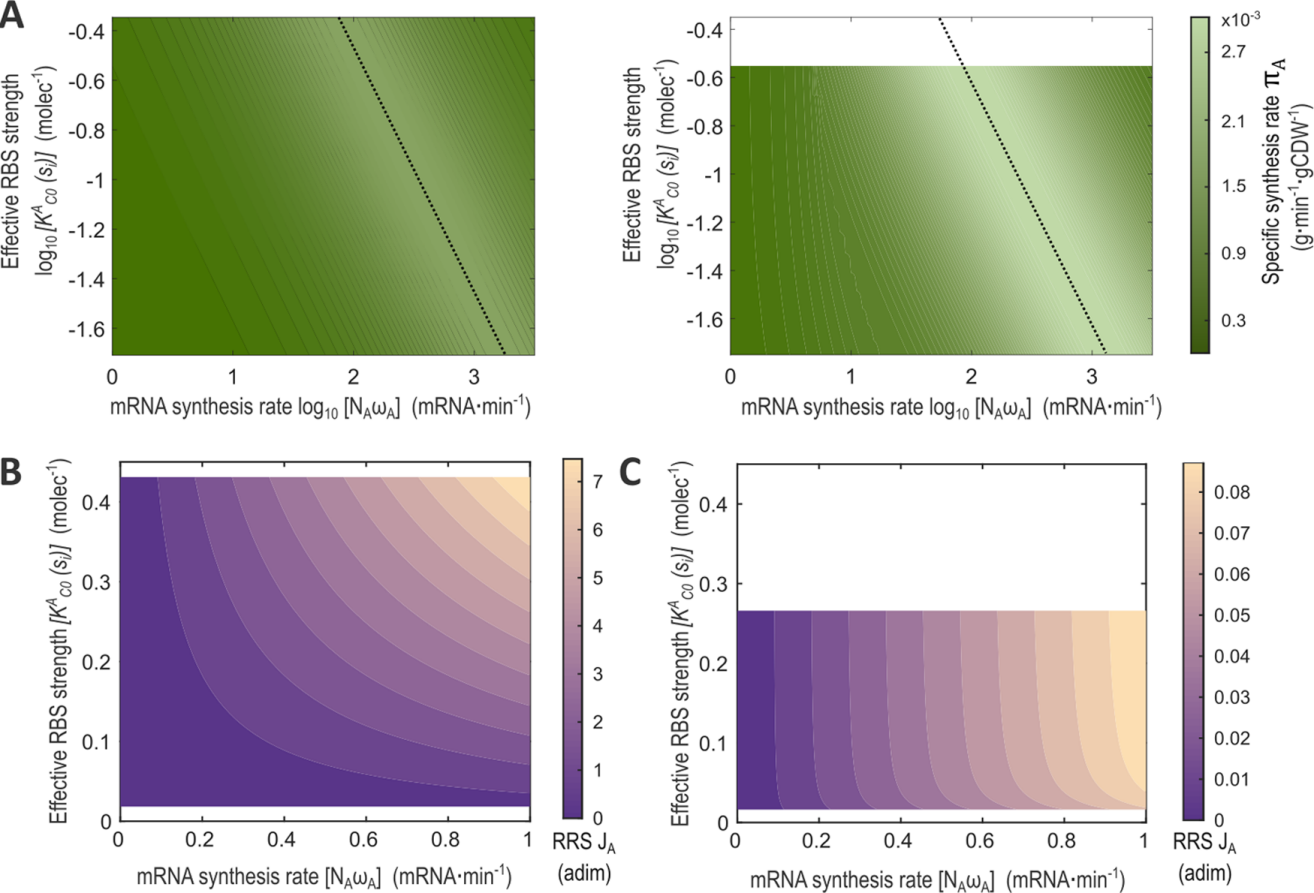
Effect of varying the mRNA synthesis rate and the effective RBS strength on (A) the specific synthesis rate of protein A and (B, C) the resources recruitment strength (*J*_A_) for different substrates. (B) Low substrate scenario ν_t_(*s*_i_) = 720 min^–1^. (C) High substrate scenario ν_t_(*s*_i_) = 1260 min^–1^. The value of *J*_A_ was evaluated for the full range of RBS values (*K*_C^0^_^A^(*s*_i_)) and a representative range of promoter values (*N*_A_ω_A_), with *E*_mA_ and *d*_mA_ equal to endogenous ribosomal values (without loss of generality).

To understand the differential role of RBS and promoter strengths, we first evaluated the dependence of the specific protein mass synthesis rate of an exogenous protein A on the mRNA synthesis rate and the effective RBS strength as a function of the substrate. Figure 6A shows the results for two representative substrate levels: low substrate ν_t_(*s*_i_) = 720 min^–1^ (left) and high substrate ν_t_(*s*_i_) = 1260 min^–1^ (right). The maximum protein synthesis rates (black dashed lines) are located at different places in the design space. Increasing the substrate had the effect of increasing the spMSR (the right plot is whiter than the left one). In addition, the optimal synthesis rate moved to the right, i.e., for the same mRNA synthesis rate, a higher effective RBS was required to reach the optimum. This implies that a cell configured to obtain the optimum protein synthesis rate for some substrate level will become suboptimal when changing the substrate level.

The resources recruitment strength (RRS) explains this differential effect of RBS and promoter strength on protein expression. For a given protein, its RRS eq 3 is directly proportional to the mRNA synthesis rate. Figure 6B,C shows that the mRNA synthesis rate effectively modifies the RRS value regardless of the substrate or the growth rate. As the mRNA synthesis rate increases (displacement to the right in the *x*-axis) the value of the RRS increases. Therefore, tuning the promoter strength implies tuning the RRS level without affecting the RRS sensitivity to changes in the substrate, the growth rate or the changing availability of free ribosomes.

Different from the promoter strength, the RBS strength determines the sensitivity of the resources recruitment strength to changes in the substrate. It has two different effects on the value of the RRS. The first effect is related to the definition of the RBS in eq 5. It depends on the association–dissociation rate constants *K*_b_^*k*^ and *K*_u_^*k*^ and indirectly on the substrate through *K*_e_(*s*_i_). For a given substrate *s*_i_, there is a set of infinite combinations of *K*_b_^*k*^ and *K*_u_^*k*^ that might provide the same RBS strength level. This causes the strength of the RBS to vary with changes in the substrate so that it decreases as the substrate increases. However, the RBS strength (and therefore the RRS value) with a high dissociation constant rate *K*_u_^*k*^ ≫ *K*_e_(*s*_i_) will be less sensitive to changes in the substrate.

On the other hand, note, from eq 5, that the RBS strength defines the sensitivity of the RRS to the flux of free resources μ*r*. Decreasing the RBS strength will always reduce the RRS value. However, increasing the RBS strength will increase the RRS value until it eventually saturates. In particular, when *d*_m_/*K*_C^0^_^*k*^ ≪ μ*r*, the RRS eq 5 becomes
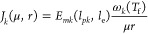
21in this case, the RRS value becomes independent of the RBS strength. Thus, there is a maximum RRS value that can be obtained by increasing the RBS strength. Figure 6B shows that for low substrate availability, the RBS can increase and yet the RRS value decrease, and Figure 6C shows the saturating effect of increasing the RBS strength for a high substrate.

For exogenous protein-coding genes, the situation is different depending on whether they do add or not a relevant burden on the cell. In case the exogenous genes do not overload the cell, the expression patterns will be the same as those for the endogenous genes analyzed above. In case the exogenous genes impose an important burden on the cell, the effects of RBS and the promoter change. In this case, μ*r* will be very small and the differential effect of the promoter and RBS strengths is partly lost. In this overloaded scenario, the RRS can be approximated as
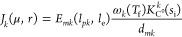
22Therefore, the RRS only depends on the substrate through the substrate-dependent value of the RBS strength. This causes the RBS–promoter strength strategies to become less differentiated. Yet, it is still possible to apply the analysis above for the distribution of mass fractions as a function of the RRS. Thus, the strong-promoter weak-RBS strategy will allow us to have RRS whose value is less sensitive to changes in the substrate compared to the weak-promoter strong-RBS one, as observed in Figure 1D.

## 3 Discussion

Our model defined the gene resources recruitment strength as the key functional coefficient that explains the distribution of resources among the host–circuit and the relationship between the use of these resources and cell growth. The RRS generalizes similar proposals in the literature, allowing us to analyze not only scenarios with high cell burden but also scenarios where the competition for cell resources does not overload the cell extremely. Conversely from the resource demand coefficient defined in ref (4), where the resource limitation effect is local, we considered that the cell resources (ribosomes) are accessible to all genes in the cell, so exogenous and endogenous host genes compete to recruit cellular resources. The assumption of constant growth rate, constant total number of ribosomes, and highly overloaded cell in ref (4) implies a static resource demand coefficient that is independent of the availability of free resources or the growth rate. This assumption is equivalent to the overloaded scenario in our model with RRS given in expression 22. However, our RBS strength does depend on the substrate. Therefore, our model can be used in scenarios where the demand on resources changes over time since the RRS explicitly captures the mass distribution dependence on cellular growth and substrate availability.

The RRS of a gene plays an important role in the value of the specific protein mass synthesis rate. Note, from eq 19, that RRS and the spMSR are related. The specific mass synthesis rate is essentially a function of the ratio between the RRS of the gene of interest and the total sum of RRSs of the cell. Therefore, it provides information about the resources that the gene of interest is capturing and sharing with other cell components to get expressed. In this sense, spMSR is a context-dependent magnitude that requires knowledge of the spMSRs of the remaining genes. The resources recruitment strength is somewhat a more fundamental characterization of a protein-coding gene than the specific protein mass synthesis rate. It is kind of a context-dependent intrinsic magnitude. Its shape only depends on the gene characteristics. Its actual value is only defined by the generic flux of free resources μ*r* and the effect of the substrate availability (which may integrate the nutrient quality) on the effective RBS strength. Therefore, the RRS measures the intrinsic avidity of a given protein-coding gene for cell resources.

Interestingly, the spMSR, i.e., the mass synthesis rate of a given protein per cell mass can be related to the definition of capacity as proposed in refs (29) and (30). There, a cell capacity monitor is implemented by including the constitutive expression of a GFP gene and determining capacity as the GFP production rate per cell of their capacity monitor. Both concepts, capacity and spMSR, are not the same but are related. In ref (30), the authors show the existence of a critical capacity. Our results, as seen in Figure 5A,B, also showed an upper bound or “critical” spMSR as a function of the mRNA synthesis rate (mRNA·min^–1^). The existence of this critical spMSR is not directly related to energy limitation, but it is the result of the peptide optimal allocation for building blocks (aas) to synthesize either a given (possibly exogenous) protein or more ribosomes. Indeed, energy limitations will indirectly affect the critical capacity value insofar as they interfere with the flow of building blocks to build up the peptide chains. From the perspective of energy as a resource, our model implicitly incorporates this concept as a fundamental part of the substrate. That is, all of the resources needed by the cell eventually come from the substrate. Consequently, our model captures this substrate–energy interaction and it is quantified by the resources recruitment strength and the substrate-dependent effective RBS strength. This approach differs from others such as ref (13), where energy is modeled explicitly after defining additional gene expression thresholds and a sigmoidal transcription/translation dependence on the energy levels.

The results obtained with our model were relevant both for the analysis of the native host cell, i.e., without exogenous protein-coding genes, and for the case of having a strain hosting exogenous protein-coding genes.

In the first case, we showed that endogenous ribosomal and nonribosomal genes clearly differ in their average resources recruitment strength and, therefore, in their average requirement for cell resources. The ribosomal proteins, essential for the cell and continuously being expressed, have higher RRS values than the nonribosomal ones. Moreover, its range of variation over the ribosomal proteins was much lower than for nonribosomal ones. This result was not fundamentally determined by the lengths of the coded proteins and is consistent with the fact that to great extent all ribosomal proteins are equally important for the cell. Transcription and translation are energetically expensive processes. It is usually accepted that around 60% of genes are expressed in standard laboratory conditions at any one time in *E. coli*, with only a small fraction making up a large percentage of the total protein. The cumulative sum of the maximum resources recruitment strength gave a good estimation of the percentage of genes expressed at any time. This is consistent with the fact that ribosomal genes are continuously needed for the cell so they are continuously expressed. On the contrary, nonribosomal genes are regulated to be expressed only when they are required. This also explains the very low RRS values obtained for them and reflects these genes are down-regulated most of the time.

It is known that weakly expressed endogenous genes exhibit low RNA polymerase (RNAP)/ribosome ratios, while strongly expressed genes have higher RNAP/ribosome ratios, as this is metabolically efficient.^11^ Our model predicted that it is not possible to achieve high expression and high robustness with respect to the resources recruitment strength by only adjusting the RBS strength. There is a trade-off among protein expression, RBS strength, robustness, and flux of free resources. The RBS strength sets the sensitivity of the resources recruitment strength with respect to the flux of free resources. Thus, strong RBSs were predicted to be associated with resources recruitment strengths more sensitive to variations in the flux of free resources (i.e., at different growth rates) while weak RBSs provide robustness with respect to the growth rate. This defines how much of a given protein (e.g., ribosomal or nonribosomal) will be expressed at different growth rates.

This trade-off was consistent with the estimated values of the average transcription rates and RBS strengths we obtained for the cell endogenous ribosomal and nonribosomal genes. We found that the low RBS strength and high transcription rate of ribosomal genes make their resources recruitment strength robust with respect to changes in the flux of free resources with growth rate. On the contrary, for nonribosomal genes, our model predicted an average high RBS strength and a low transcription rate expression strategy. This differential strategy allowed us to explain the relative mass fractions distributions of endogenous ribosomal and nonribosomal proteins as a function of growth rate. Thus, the differential expression strategies in *E. coli* encode the mass distribution of ribosomal and nonribosomal proteins for varying growth rates. Our model suggested that the cell achieves a fairly constant absolute expression of nonribosomal proteins using a high RBS strength to express them. On the other hand, the cell uses much weaker RBSs to express the ribosomal proteins. This way, the value of the total ribosomal RRS remains mostly constant with respect to the nonribosomal one. As a consequence, the absolute expression of ribosomal proteins increases with growth rate.

The results were applicable to the expression of exogenous protein-coding genes. For a given ratio of RRSs, increasing the expression of exogenous genes decreases the growth rate thereby reducing the absolute mass of endogenous proteins. However, the mass of exogenous proteins accumulates in the cell, which allows the total mass of the cell to increase even if the growth rate decreases. Two extreme cases can be differentiated: either the exogenous genes imposing negligible loading on the cell or strongly overloading it. In the first case, the exogenous proteins behave in an equivalent way to the endogenous ones. Therefore, all of the results obtained for the last are applicable. This situation is of interest in situations like, e.g., when designing gene synthetic circuits for feedback regulation of enzymes expression in metabolic pathways. In this case, one of the goals is that the exogenous circuit does not load the cell in excess, as this will affect the overall performance of the regulated pathway. In the highly overloaded scenario, the RRS no longer depends on the flux of free resources. This causes a diminished differential effect of the RBS and promoter strength. Yet, the different sensitivity of the RBS to the available substrate as a function of its strength still has consequences in scenarios with variable substrate. In between, the definition of the RRS allows us to consider a wide range of scenarios with varying cell burden.

## 4 Conclusions

In this work, we have presented a small-size model of gene expression dynamics accounting for host–circuit interactions. The good agreement between the predictions of our model and experimental data highlights the relevance of the cellular resources recruitment strength defined in our model as a key functional coefficient. Our resources recruitment strength coefficient allows us to explain the distribution of resources between the host and the genes of interest. Additionally, it shapes the relationship between the use of resources, cell growth, and protein productivity. This functional coefficient explicitly considers the interplay between the flux of available free resources and lab-accessible gene expression characteristics. In particular, the promoter and RBS strengths.

Though we only considered *E. coli*, our findings can be extrapolated to other microorganisms, and the model can be easily fitted using a small amount of experimental data of the host cell.

Among other predictions, the model provides insights into how the differential role of promoter and RBS strengths in protein expression may have evolved in *E. coli* and other microorganisms to encode the mass distribution between ribosomal and nonribosomal proteins as a function of cell growth rate. Weak transcription and strong translation and the complementary strong transcription and weak translation emerge as two potentially equally optimal strategies in the expression space but with different characteristics from the point of view of the sensitivity of the specific synthesis rate of the expressed protein to variations in the cell growth. The capacity of the defined resources recruitment strength functional coefficients to capture the interaction between growth, cell resources, and gene expression characteristics is reflected in the fact that the model was able to infer good predictions of the experimental distribution of the cell endogenous ribosomal and nonribosomal protein mass fractions when fitted to estimate the cell specific growth rate.

The model also explains some of the phenomena typically encountered when building protein expression systems in synthetic biology. Thus, for instance, it explains the limited effect that increasing the RBS strength has to increase the expression of a given protein of interest, saturating at high RBS strengths. Design of synthetic genetic circuits without considering the impact of host–circuit interactions results in an inefficient design process and lengthy trial-and-error iterations to appropriately tune a circuit’s expression levels.^13^ In this context, our model may also be useful for design purposes in synthetic biology where it can be used to design the proper promoter–RBS strategy depending on the desired behavior of the genes expression as a function of growth rate. In this sense, the resources recruitment strength can be used as a context-dependent intrinsic magnitude for the standard characterization of protein-coding transcription units.

Further extensions of the model can be easily implemented. Thus, the model explicitly considers the relationship between the cell specific growth rate and the population dynamics. As a consequence, it can be integrated within a multiscale framework that considers the macroscopic extracellular dynamics of the substrate and population of cells in a bioreactor. The model only requires as input a measure of the fraction of available substrate with respect to the saturated case and predicts both the resulting cell specific cell growth rate and the mass and mass rates of the expressed proteins. This makes its integration with constraint-based models of metabolism rather straightforward. The possibility to consider expression systems using orthogonal ribosomes can also be implemented without much difficulties. All this makes the model useful in the context of model-based design of gene synthetic circuits and protein expression systems.

## 5 Methods

### 5.1 Model Parameters

Table SI.2 in Section SI.11 shows the set of parameters used in the model.

### 5.2 Estimation of the Parameters for Ribosomal and Nonribosomal Endogenous Proteins

We considered the model expressions at steady state in Section 2.1 and estimated the RBS-strength-related parameters *K*_b_^*k*^, *K*_u_^*k*^ with the transcription rates ω*_k_* with *k* = {r, nr} and the fraction Φ_m_ so that our model provided a good fit of the specific growth rate at steady state.

The only input information given to the model was the value of the peptide chain elongation rate values ν_t_(*s*_i_) as a function of growth rate obtained from ref (22). This is equivalent to feeding the model only with the available amount of substrate *s*_i_. To this end, we expressed the effective maximum translation rate as ν_t_(*s*_i_) = ν*f*(*s*_i_), where ν is the maximum attainable peptide synthesis rate (see eq 2) and *f*(*s*_i_) = *s*_i_/(*K*_sc_ + *s*_i_). Note that *f*(*s*_i_) is monotonous with the amount of intracellular substrate *s*_i_. From the experimental values of ν_t_(*s*_i_) as a function of growth rate and knowing the maximum attainable peptide synthesis rate ν (see Table SI.2), we obtained the experimental values of *f*(*s*_i_) for each growth rate. We used these to feed our model. This is tantamount to feeding the model with the substrate *s*_i_, but the value of the substrate- and host-dependent Michaelis constant *K*_sc_ needs not to be known.

Then, we fitted the model parameters using the experimental growth rate as the output to predict. So as not to penalize large errors in excess, which in our case are more prone to happen for larger values of the growth rate, we minimized the sum over the experimental data points of the absolute prediction error of the growth rate
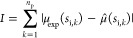
We considered *N*_r_ = 57; and *N*_nr_ = 1735, corresponding to the number of genes that explain 99% of the cumulative sum of the resources recruitments strengths for ribosomal and nonribosomal proteins, respectively (see Section 2.2). We also considered the average mRNA degradation rates *d*_m,r_ = 0.16 min^–1^ and *d*_m,nr_ = 0.2 min^–1^ (see Table SI.2). Using the value of ν in Table SI.2 and the range of *l*_e_ obtained in Section SI.14, we estimated .

Moreover, the values of the association and dissociation rates of the ribosome to the RBS, *K*_b_^*k*^ and *K*_u_^*k*^, may vary in a large range. Values *K*_b_^*k*^ ⊂ [3, 15] molecule^–1^·min^–1^ are found in the literature (see Table SI.2). We used a conservative upper bound *K*_b_^max^ = 10 molecule^–1^ for the search space, considering binding is diffusion controlled. From the literature, we also considered a search range for the dissociation rate *K*_u_^*k*^ ⊂ [3, 135] min^–1^. Overall, these estimates gave us a range *K*_C^0^_^*k*^ ⊂ [0.02, 0.2] molecule^–1^ for the effective RBS strength under the assumption of intracellular substrate saturation. We ran 200 instances of the parameter fitting algorithm using the global optimization software MEIGO^31^ (available at http://gingproc.iim.csic.es/meigo.html) and obtained the weighted mean of the 25 runs achieving the best minimum value for the sum over the experimental data points of the absolute growth rate prediction error. The resulting average best-fit estimated parameters are given in Table 1.

## Supporting Information Available

Biochemical pseudoreactions of the model; assumptions and detailed derivation of the burden-aware dynamic model; estimation of model parameters from experimental data and additional results relating the spMSR with the substrate availability and the cell growth rate as a function of the expression space; and software code for the model computational simulations and parameters optimization (PDF)
