# Supplementary material for: RBS and Promoter Strengths Determine the Cell-Growth-Dependent Protein Mass Fractions and Their Optimal Synthesis Rates
Source: ACS Synth Biol. 2021 Nov 12;10(12):3290–303. doi: 10.1021/acssynbio.1c00131 (PMC8689641; doi:10.1021/acssynbio.1c00131)
Supplement: Supplementary file 1 — sb1c00131_si_001.pdf [file sb1c00131_si_001.pdf]

Supporting Information. RBS and promoter strengths determine the cell growth-dependent protein mass fractions and their optimal synthesis rates.

Fernando N. Santos-Navarro[a], Alejandro Vignoni[a], Yadira Boada[a], Jesús Picó[a]

[a]*Synthetic Biology and Biosystems Control Lab, Institut d'Automàtica i Informàtica Industrial, Universitat Politècnica de València, Camí de Vera s/n, 46022, Valencia, Spain. Corresponding author: J. Picó: jpico@ai2.upv.es*

Contents

SI.1 Modeling gene expression 1

SI.2 Dynamics of the number of mature available ribosomes 7

SI.3 Relating free and available ribosomes 8

SI.4 Fractions of bound and actively translating ribosomes with respect to the available mature ones 9

SI.5 Obtaining the cell specific growth rate 10

SI.6 Cell specific growth rate and population dynamics 12

SI.7 Relationship between growth rate and cell mass 13

SI.8 Average host dynamics and steady state balanced growth. 15

SI.9 Synthesis rate of exogenous proteins and interaction with the host cell 17

SI.10 Model parameters 18

SI.11 Estimation of the fractions Φ_t^h and Φ_b^h 18

SI.12 Evaluation of the maximum resources recruitment strength. 21

SI.13 Estimation of the number of free ribosomes in the cell 21

SI.14 Estimated fractions of ribosomes 25

SI.15 Effect of substrate availability of growth rate and specific synthesis rate 26

SI.16 Software code and data 26

SI.1. Modeling gene expression

In this section we obtain the the dynamics for the polysomic expression of a protein-coding gene in a bacterial cell. We introduce the *resources recruitment strength* (RRS), a dimensionless function that expresses the capacity of a protein-coding gene to engage cellular resources to get expressed. The RRS serves as basis for all posterior analysis of the partitioning of the cell mass between ribosomal and non-ribosomal endogenous proteins, the effects caused by the expression of exogenous protein-coding genes, and the roles played by the RBS and promoter strengths.

In our model, we consider a set of basic assumptions:

1. Transcription dynamics is fast enough as compared to translation so it can be considered at quasi-steady state (QSS).
2. The main resources-dependent process in protein expression is translation. Therefore:
   (a) only ribosomes are considered as limiting shared resource required for protein expression. RNA polymerase is not considered explicitly.
   (b) the effective translation rate is assumed to depend on the availability of intracellular substrate. This is implicitly considering that building the polypeptide protein chain is the limiting energy-consuming process in the cell. We do not explicitly consider the catabolic conversion of substrate into amino acid building blocks.

3. Several ribosomes (a polysome) may translate a single messenger RNA (mRNA) simultaneously.
4. We identify a transcriptional unit by its promoter.

With these assumptions in mind, we modeled the expression of a given gene expressing the protein p_k by means of the set of pseudo-reactions 1.

$$\begin{aligned}
\mathrm{g_k} &\xrightarrow{\omega_k(T_f)} \mathrm{g_k} + \mathrm{m_k} \\
\mathrm{m_k} + \mathrm{r} &\underset{K_u^k}{\overset{K_b^k}{\rightleftharpoons}} \mathrm{C_{k^0}} \\
\mathrm{C_{k^0}} &\xrightarrow{K_e(s_i)} \mathrm{C_{k^1}} + \mathrm{m_k} \\
\mathrm{C_{k^1}} &\xrightarrow{K_k(s_i)} \mathrm{p_k} + \mathrm{r} \\
\mathrm{C_{k^1}} &\xrightarrow{K_e(s_i)} \mathrm{C_{k^2}} \\
&\vdots \\
\mathrm{C_{k^{j-1}}} &\xrightarrow{K_k(s_i)} \mathrm{p_k} + \mathrm{r} \\
\mathrm{C_{k^{j-1}}} &\xrightarrow{K_e(s_i)} \mathrm{C_{k^j}} \\
&\vdots \\
\mathrm{C_{k^{n_r-1}}} &\xrightarrow{K_e(s_i)} \mathrm{C_{k^{n_r}}} \\
\mathrm{C_{k^{n_r}}} &\xrightarrow{K_k(s_i)} \mathrm{p_k} + \mathrm{r} \\
\mathrm{m_k} &\xrightarrow{d_{mk}+\mu} \emptyset \\
\mathrm{p_k} &\xrightarrow{d_k+\mu} \emptyset \\
\mathrm{C_{k^j}}_{\mathrm{j=0\ldots n_r}} &\xrightarrow{\mu} \emptyset
\end{aligned} \tag{1}$$

where the species involved are:

g_k : free copy of the promoter, ie. of the gene transcriptional unit.

m_k : free ribosome binding site (RBS), ie. mRNA copy with its RBS free.

r : free ribosome.

C_{k^0} : complex formed by a ribosome bound to the RBS in a mRNA

C_{k^j} : j-th translating complex formed by j ribosomes simultaneously translating a mRNA copy with freed RBS. Each C_{k^j} species gives rise to a peptide chain which is in different stages of production.

p_k : protein abundance

s_i : intracellular substrate (molecules)

and the specific reaction rates stand for:

$\omega_k(T_f)$: transcription rate. It may be a function of one or several transcription factors (TF) and may include the gene copy number.

K_b^k : association rate constant between a free ribosome and the RBS.

K_u^k : dissociation rate constant between a free ribosome and the RBS.

$K_e(s_i)$: translation initiation rate. constant.

$K_k(s_i)$: translation elongation rate constant.

d_{mk} : mRNA degradation rate constant.

d_k : protein degradation rate constant.

μ : cell specific growth rate.

For a protein of length l_{pk} amino acids, we denote the mRNA length:

$$l_{mk} = l_e + l_{pk} \tag{2}$$

where $1/l_e$ is the ribosomes density, with l_e expressed as equivalent number of codons. We consider the effective RBS length to be the same as l_e. The length of the protein is denoted as l_{pk}. Thus, up to n_r ribosomes can simultaneously be translating a single copy of mRNA, with $n_r = l_{pk}/l_e$, and an additional ribosome is bound to the RBS.

We consider the effective rates constants $K_e(s_i)$ and $K_k(s_i)$ at which the ribosome glides through the RBS and the remaining mRNA nucleotides respectively. Thus, we consider:

$$K_e(s_i) = \frac{\nu_t(s_i)}{l_e}\ ,\ K_k(s_i) = \frac{\nu_t(s_i)}{l_{xk}} \tag{3}$$

with

$$\nu_t(s_i) = \frac{\nu s_i}{K_{sc} + s_i} \tag{4}$$

where ν is the maximum attainable translation rate (peptide synthesis rate) and K_{sc} is a Michaelis-Menten parameter related to the cell substrate uptake and catabolic capacity. As a first approximation, we consider that ν is organism dependent but does not depend on the sequence of nucleotides, and K_{sc} is organism and substrate dependent but does not depend on the nucleotides sequence either.

The translating complexes $C_{k^{j-1}}$ are pseudo-species modeling the process of parallel translation. They can loosely be identified with each of the chains of amino acids under formation plus its associated ribosome. The first one, C_{k^0}, represents the ribosome bound to the RBS. Notice with rate $K_e(s_i)$ the ribosome bound to the RBS, forming C_{k^0}, advances to the next ribosome occupancy slot, generating the translating complex C_{k^1} and freeing the RBS so a new ribosome can enter in the queue. In turn, the displacement of the ribosomes in the queue by one occupancy generates the translating complexes C_{k^j} from the previous complex $C_{k^{j-1}}$. Thus, each C_{k^j} species gives rise to a peptide chain which is in different stages of production. Finally, each parallel translating complex C_{k^j} generates a protein with rate $K_k(s_i)$ and frees its bound ribosome. Recall the translating complexes can be identified with the ensemble formed by each of the chains of amino acids under formation and their respective associated ribosomes. This way, the queue dynamics of the ribosomes advancing along the mRNA is decoupled from the protein building ones, thus getting a continuous approximation of the polysomic process of translation. This continuous approximation results in an exponential distribution of the amounts of pseudo-complexes C_{k^j}.

Figure SI.1 depicts the process. The amounts of the pseudo-complexes fulfill $C_{k^j} < C_{k^{j-1}}$ (see equation (9)). Thus, starting from C_{k^0}, at each time instant a fraction of it remains and a smaller fraction becomes C_{k^1} because of the continuous shuffling up. At the next differential time instant, on the one hand a fraction of C_{k^1} remains and a smaller fraction becomes C_{k^2} and, on the other hand, a fraction of the C_{k^0} which remained in the previous time step remains and a smaller fraction becomes C_{k^1}. This process of generation of the amounts of pseudo-complexes takes place at each differential time instant, eventually resulting in an exponential distribution of the amounts of pseudo-complexes C_{k^j}. In parallel (i.e. simultaneously) to the process above, each of the pseudo-complexes C_{k^j} generates a peptide chain through the pseudo-reactions $\mathrm{C_{k^j}} \xrightarrow{K_k(s_i)} \mathrm{p_k} + \mathrm{r}$. As a result the protein synthesis rate $K_k(s_i)\sum_{j=1}^{n_r} C_{k^j}$ (see equation (5)) will effectively be weighting the amount of proteins being synthesized at each of their synthesis stages. Recall again that the pseudocomplexes C_{k^j} can loosely be identified with each of the chains of amino acids under formation plus its associated ribosome. The less abundant ones at the later stages of the peptide chain synthesis (close or at $C_{k^{n_r}}$) are weighted much less than the more abundant shorter ones at earlier stages. This way, the evaluation of the protein synthesis rate takes into account an approximation of the distribution profile of the lengths of the peptide chains being synthesized by the ribosomes along the transcript.

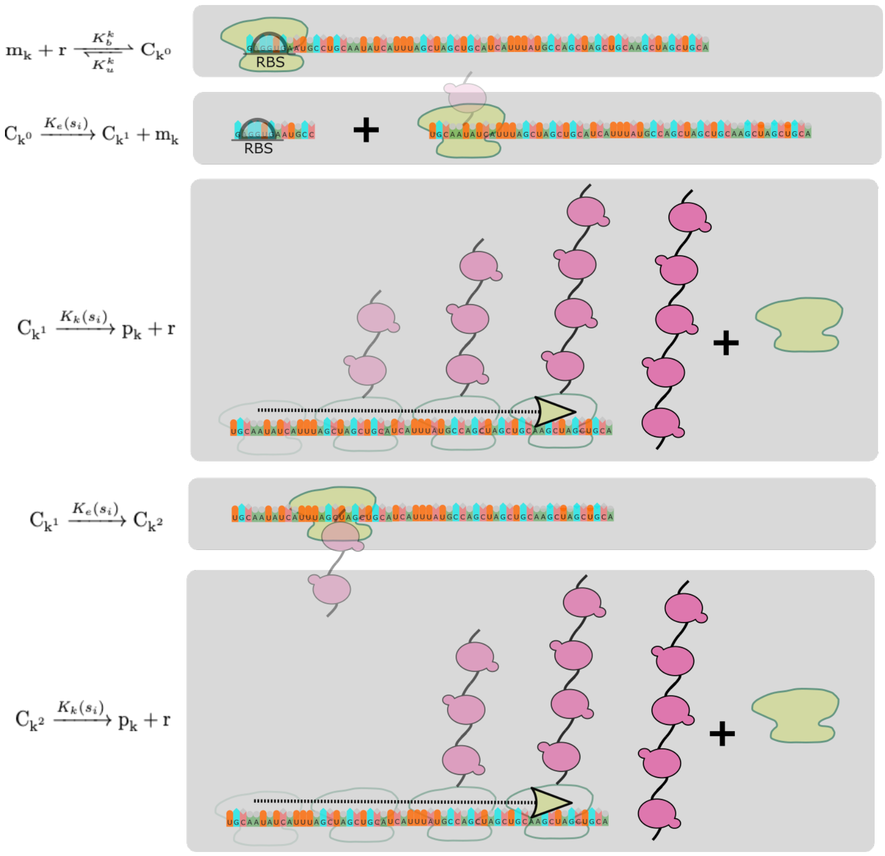

Figure SI.1. Modeling polysomic translation. From top to bottom. **(1)** For a k-th protein-coding gene, a free ribosome binds to m_k, a mRNA copy with its RBS free, producing the initial pseudocomplex C_{k^0}: an avatar for a transcript with a ribosome bound to its RBS. **(2)** The ribosome shuffles up one space with rate constant equal to the translation initiation one $K_e(s_i)$ leaving the same mRNA copy with a free RBS, m_k. In addition, there will be a ribosome placed at the first slot of the transcript protein coding region This ensemble is represented by the pseudocomplex C_{k^1}. Thus, though physically there is only one transcript copy with a free RBS and a ribosome placed at the first slot of the transcript coding region, we model this situation as having two pseudospecies: m_k and C_{k^1}. The mRNA is again free to be bound by a new free ribosome, allowing new ribosomes to continually load into the system. Thus, ribosomes enter the translation process through the continuous "cycling" between the second and third pseudo-reactions in SI equation (1) and their shuffling up through the chain pseudo-reactions $\mathrm{C}_{\mathrm{k}^{j-1}} \xrightarrow{K_e(s_i)} \mathrm{C}_{\mathrm{k}^{j}}$. **(3)** C_{k^1} becomes an independent pseudocomplex consisting of a ribosome placed at the first slot of the mRNA coding region and gliding along the transcript with translation elongation rate constant $K_k(s_i)$. This virtual representation allows to consider the pseudoreaction $\mathrm{C}_{\mathrm{k}^1} \xrightarrow{K_k(s_i)} \mathrm{p}_\mathrm{k} + \mathrm{r}$. Eventually, one copy of the protein is synthesized and the ribosome is freed. **(4)** When the ribosome has initiated translation in C_{k^1} all other ribosomes will shuffle up one space in the physical mRNA. In our model we shuffle up the pseudocomplexes C_{k^j}. Thus, C_{k^1} becomes C_{k^2} at rate $K_e(s_i)$ at which the entering free ribosome into the system "pushes" the queue to shuffle up. **(5)** A second parallel translation $C_{k^2} \rightarrow p_k + r$ takes place. Both C_{k^1} and C_{k^2} simultaneously synthesize a protein and eventually free their bound ribosomes (one per complex).

Next, we apply mass action kinetics to obtain the dynamic balances for the copy number of each species

in the model. This way, we have:

$$
\begin{aligned}
\dot{m}_k &= \omega_k(T_f) - K_b^k m_k r + K_u^k C_{k^0} + K_e(s_i) C_{k^0} - (d_{mk} + \mu) m_k \\
\dot{C}_{k^0} &= K_b^k m_k r - K_u^k C_{k^0} - K_e(s_i) C_{k^0} - \mu C_{k^0} \\
\dot{C}_{k^1} &= K_e(s_i) C_{k^0} - K_e(s_i) C_{k^1} - K_k(s_i) C_{k^1} - \mu C_{k^1} \\
&\vdots \\
\dot{C}_{k^{j-1}} &= K_e(s_i) C_{k^{j-2}} - K_e(s_i) C_{k^{j-1}} - K_k(s_i) C_{k^{j-1}} - \mu C_{k^{j-1}} \\
&\vdots \\
\dot{C}_{k^{n_r}} &= K_e(s_i) C_{k^{n_r-1}} - K_e(s_i) C_{k^{n_r}} - K_k(s_i) C_{k^{n_r}} - \mu C_{k^{n_r}} \\
\dot{p}_k &= K_k(s_i) \sum_{j=1}^{n_r} C_{k^j} - (d_k + \mu) p_k
\end{aligned}
\tag{5}
$$

Recall we assume that transcription dynamics is fast enough as compared to translation so it can be considered at quasi-steady state (QSS). We also assume the binding-unbinding dynamics to form the translation complexes C_{k^j} are fast enough so that we can also consider the number of each of the complexes quickly reaches steady state. Therefore, from $\dot{m}_k = 0$ and $\dot{C}_{k^j, j=1\ldots n_r} = 0$ we get:

$$
C_{k^0} = \omega_k(T_f) \frac{1}{\frac{d_{mk}+\mu}{\frac{K_b^k}{K_u^k + K_e(s_i) + \mu}} + \mu r} r \tag{6}
$$

and

$$
C_{k^j} = \frac{K_e(s_i)}{K_e(s_i) + K_k(s_i) + \mu} C_{k^{j-1}} \quad , \quad j = 1 \ldots n_r \tag{7}
$$

In practice, $d_{mk} \gg \mu$ and $k_u^k \gg \mu$ (see Table SI.2). Therefore the magnitude of the specific growth rate μ can be neglected with respect to both the mRNA degradation rate constant d_{mk} and the sums $K_u^k + K_e(s_i)$ and $K_e(s_i) + K_k(s_i)$ respectively so that we can approximate:

$$
C_{k^0} \approx \omega_k(T_f) \frac{1}{\frac{d_{mk}}{K_{C^0}^k} + \mu r} r \tag{8}
$$

and

$$
C_{k^j} \approx \frac{K_e(s_i)}{K_e(s_i) + K_k(s_i)} C_{k^{j-1}} \quad , \quad j = 1 \ldots n_r \tag{9}
$$

where we have defined:

$$
K_{C^0}^k(s_i) \triangleq \frac{K_b^k}{K_u^k + K_e(s_i)} \tag{10}
$$

Notice $K_{C^0}^k$ is directly related to the RBS strength.

Now, the dynamics for the abundance of the protein p_k can be obtained from the equations (5) and (9) as:

$$
\begin{aligned}
\dot{p}_k &= K_k(s_i) \sum_{j=1}^{n_r} C_{k^j} - (d_k + \mu) p_k \\
&= K_k(s_i) a \left[1 + a + \ldots + a^{n_r - 1}\right] C_{k^0} - (d_k + \mu) p_k
\end{aligned}
\tag{11}
$$

with $a \triangleq \frac{K_e(s_i)}{K_e(s_i) + K_k(s_i)}$.

Notice the geometric sum $1 + a + \ldots + a^{n_r-1}$, with $a < 1$, gives:

$$
\begin{aligned}
K_k(s_i) a \left[1 + a + \ldots + a^{n_r-1}\right] &= K_k(s_i) a \frac{1 - a^{n_r}}{1 - a} \\
&= K_e(s_i) \left[1 - \left(\frac{K_e(s_i)}{K_e(s_i) + K_k(s_i)}\right)^{n_r}\right]
\end{aligned}
\tag{12}
$$

Using the definitions (3), notice $a = \frac{l_{pk}}{l_e + l_{pk}} = \frac{l_{pk}}{l_{mk}}$ so we get the expression:

$$K_k(s_i)a\left[1 + a + \ldots + a^{n_r - 1}\right] = K_e(s_i)\left[1 - \left(\frac{l_{pk}}{l_{mk}}\right)^{\frac{l_{pk}}{l_e}}\right] \tag{13}$$

where we have taken into account that the maximum number of ribosomes bound to active translating complexes $C_{k^j, j=1\ldots n_r}$ simultaneously translating a mRNA molecule is $n_r = l_{pk}/l_e$, and we assume this maximum is always reached.

Recall we have assumed that transcription is not a limiting process, so we can express the effective transcription rate:

$$\omega_k(T_f) = \frac{\eta}{l_{mk}} F_k(T_f) \tag{14}$$

where η (codons/min) is the maximum transcription speed and $F_k(T_f)$ is the transcription characteristic function that may depend on one or several transcription factors. By default, we assume the gene copy number c_{nk} is one. If this is not the case, the effective transcription rate $\omega_k(T_f)$ must be multiplied by c_{nk}. Notice in this case the transcription characteristic function $F_k(T_f)$ may depend on the gene copy number as described in [23].

As commented in our preliminary assumptions, we do not model competition for RNA polymerases, which would also affect the effective transcription rate. Yet, notice that, even if there are no cognate transcription factors associated, the term $F_k(T_f)$ can be used to account for competition for RNA polymerases preventing the effective transcription from proceeding at its maximum rate $\frac{\eta}{l_{mk}}$, sequence-dependent affinity of the promoter for the RNA polymerases (promoter strength) and the effect of nucleotides usage on the transcription speed. In summary, the term $F_k(T_f)$ can be used to accommodate aspects affecting transcription so that $\omega_k(T_f)$ is the effective transcription rate.

Thus, the dynamics for protein expression become:

$$\begin{aligned}
\dot{p}_k &= K_e(s_i)\left[1 - \left(\frac{l_{pk}}{l_{mk}}\right)^{\frac{l_{pk}}{l_e}}\right] C_{k^0} - (d_k + \mu)p_k \\
&= K_e(s_i)\left[1 - \left(\frac{l_{pk}}{l_{mk}}\right)^{\frac{l_{pk}}{l_e}}\right] \frac{\eta}{l_{mk}} F_k(T_f) \frac{1}{\frac{d_{mk}}{K^k_{C^0}} + \mu r} r - (d_k + \mu)p_k \\
&= \frac{\nu}{l_e} \frac{s_i}{K_{sc} + s_i}\left[1 - \left(\frac{l_{pk}}{l_{mk}}\right)^{\frac{l_{pk}}{l_e}}\right] \omega_k(T_f) \frac{1}{\frac{d_{mk}}{K^k_{C^0}} + \mu r} r - (d_k + \mu)p_k
\end{aligned} \tag{15}$$

We now define the ribosomes density related term:

$$E_{mk}(l_{pk}, l_e) \triangleq \frac{l_{pk}}{l_e}\left[1 - \left(\frac{l_{pk}}{l_{mk}}\right)^{\frac{l_{pk}}{l_e}}\right] = \frac{l_{pk}}{l_e}\left[1 - \left(1 - \frac{l_e}{l_{mk}}\right)^{\left(\frac{l_{mk}}{l_e} - 1\right)}\right] \tag{16}$$

Figure SI.2 shows the values of $E_{mk}(l_{pk}, l_e)$ as a function of the protein length l_{pk} for different values of l_e. As seen, $E_{mk}(l_{pk}, l_e)$ can be accurately approximated as the linear function of l_{pk}/l_e:

$$E_{mk}(l_{pk}, l_e) \approx 0.62 \frac{l_{pk}}{l_e} \tag{17}$$

We further define the *resources recruitment strength* (RRS) functional parameter $J_k(\mu, r)$:

$$J_k(\mu, r) \triangleq E_{mk}(l_{pk}, l_e)\omega_k(T_f) \frac{1}{\frac{d_{mk}}{K^k_{C^0}} + \mu r} \tag{18}$$

Notice the *resources recruitment strength* is a dimensionless function that expresses the capacity of the k-th protein-coding gene to engage cellular resources to get expressed. It explicitly depends on:

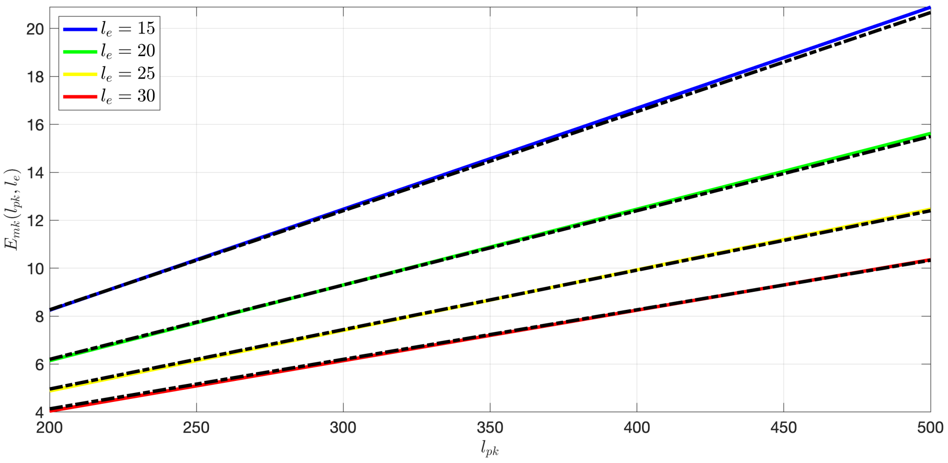

Figure SI.2. Function $E_{mk}(l_{pk}, l_e)$ as a function of the protein length l_{pk} for different values of l_e and their corresponding linear approximations $E_{mk}(l_{pk}, l_e) = 0.62\frac{l_{pk}}{l_e}$.

- the gene expression characteristics:
  - mRNA transcription rate $\omega_k(T_f)$ and degradation rate constant d_{mk}
  - RBS strength-related parameter $K^k_{C^0}(s_i)$
- and the availability of cell resources:
  - flux of free ribosomes μr
  - ribosomes density $\frac{l_{pk}}{l_e}$ (via $E_{mk}(l_{pk}, l_e)$)

Using these definitions in equation (15) we get the expressions for the abundance dynamics of the k-th protein:

$$\begin{aligned} \dot{p}_k &= 0.62\frac{\nu_t(s_i)}{l_e}\frac{r}{\frac{d_{mk}}{K^k_{C^0}(s_i)} + \mu r}\omega_k(T_f) - (d_k + \mu)p_k \\ &= \frac{\nu_t(s_i)}{l_{pk}}J_k(\mu, r)r - (d_k + \mu)p_k \end{aligned} \tag{19}$$

SI.2. Dynamics of the number of mature available ribosomes

Next, using the results of the previous section, we obtain the dtnamics of the number of mature available ribosomes. Ribosomes are large complexes formed by both ribosomal RNA molecules and a variety of ribosomal proteins, adding up to 55 different protein species in *E. coli*. Recall we consider that translation is the main energy and resources limiting process we model. Notice the total number of ribosomes in the cell at any one time instant r_T is the sum of the mature (r_a) and inmature (r_i) ribosomes. The mature ribosomes r_a available for protein translation comprise the free ribosomes r and the ones bound to translating complexes. The bound ribosomes comprise the ones bound to translating complexes building the ribosomes themselves (r^r_b), the ones bound to the translating complexes of endogenous non-ribosomal proteins (r^{nr}_b) and the ones associated to the expression of exogenous genes (r^{exo}_b). In turn, the bound ribosomes may be either bound to the RBSs or actively elongating along the transcripts to synthesize proteins. We denote the last subset as r^r_t, r^{nr}_t and r^{exo}_t respectively. We also denote $r^h_b = r^r_b + r^{nr}_b$ as the set of *host* ribosomes bound both to ribosomal and non-ribosomal endogenous complexes. In case we are interested in a particular *strain* hosting

exogenous genes, we refer to the set of all bound ribosomes as $r_b^s = r_b^h + r_b^{\rm exo}$. In sumamry, we have:

$$\begin{aligned} r_T &= r_a + r_i \\ r_a &= r + r_b^s \\ r_b^s &= r_b^h + r_b^{\rm exo} \\ r_b^h &= r_b^r + r_b^{nr} \end{aligned} \tag{20}$$

An analogous notation is used for the actively elongating bound ribosomes, with $r_t^h = r_t^r, +r_t^{nr}$ and $r_t^s = r_t^h + r_t^{\rm exo}$.

The number of available mature ribosomes is a fraction of the total number of ribosomes, so that $r_a = \Phi_m r_T$. The fraction of active elongating ribosomes varies little in time, with an average value 0.8 [2, 3]. Therefore, we can expect that the fraction Φ_m also varies little, so that the dynamics of the total number of ribosomes and that of the number of available ribosomes are the same but for a scale factor. Next we consider the dynamics of the total copy number of ribosomes in the cell, r_T.

To get the dynamics of r_T we first consider an analogous expression to (19) for each of the proteins forming up a ribosome. If we consider the average ribosomal protein p_r, and an average ribosome composed of N_r proteins (e.g. $N_r = 55$ for *E. coli*) we define the total number of ribosomal proteins as $p_{\Sigma r} = N_r p_r$. For the average ribosomal protein p_r we have use (19) to obtain dynamics:

$$\begin{aligned} \dot{p}_r &= \frac{\nu}{l_{p_r}} \frac{s_i}{K_{sc} + s_i} E_{m,r} \omega_r \frac{1}{\frac{d_{mr}}{K^r_{C^0}} + \mu r} r - \mu p_r \\ &= \frac{\nu}{l_{p_r}} \frac{s_i}{K_{sc} + s_i} J_r(\mu, r) r - \mu p_r \end{aligned} \tag{21}$$

where we have assumed that ribosomal proteins are only subject to dilution caused by cell growth.

Then, the dynamics for the total number of ribosomal proteins can be approximated as:

$$\begin{aligned} \dot{p}_{\Sigma r} &= N_r \dot{p}_r \\ &= \frac{\nu}{l_{p_r}} \frac{s_i}{K_{sc} + s_i} N_r J_r(\mu, r) r - \mu p_{\Sigma r} \end{aligned} \tag{22}$$

Since all N_r protein species are needed to form up an individual ribosome, and considering the average ribosomal protein p_r, the total number of ribosomes is $r_T = p_{\Sigma r}/N_r$. Therefore, the dynamics of the total abundance of ribosomes r_T will be the same as those of p_r. That is:

$$\begin{aligned} \dot{r}_T &= \frac{\nu}{l_{p_r}} \frac{s_i}{K_{sc} + s_i} E_{m,r} \omega_r \frac{1}{\frac{d_{mr}}{K^r_{C^0}} + \mu r} r - \mu r_T \\ &= \frac{\nu}{l_{p_r}} \frac{s_i}{K_{sc} + s_i} J_r(\mu, r) r - \mu r_T \end{aligned} \tag{23}$$

Therefore, the dynamics of the number of mature available ribosomes is:

$$\dot{r}_a = \frac{\nu}{l_{p_r}} \frac{s_i}{K_{sc} + s_i} J_r(\mu, r) \Phi_m r - \mu r_a \tag{24}$$

SI.3. Relating free and available ribosomes

The number of free ribosomes is a measure of the cell burden, and plays a central role in the synthesis rate of proteins. Next we relate the number of free ribosomes with that of the synthesized available ones. That is, the available mature ones in the cell. This relationship will later allow to express the dynamics of the total number (alternatively, mass) of cell ribosomes and non-ribosomal proteins as a function of their interaction

Recall we had $r_a = r + r_b^s$. For each protein p_k, and using the previous results, the number of ribosomes bound to complexes involved in its translation at each time instant is given by:

$$\begin{aligned} r_b^{p_k} &= C_{k^0} + \sum_{j=1}^{n_r} C_{k^j} = \left[1 + \frac{a}{1-a}(1-a^{n_r})\right] C_{k^0} = \left[1 + E_{mk}(l_{pk}, l_e)\right] C_{k^0} \\ &= \left[1 + E_{mk}(l_{pk}, l_e)\right] \frac{1}{E_{mk}(l_{pk}, l_e)} J_k(\mu, r) r = \left[1 + \frac{1}{E_{mk}(l_{pk}, l_e)}\right] J_k(\mu, r) r \end{aligned} \tag{25}$$

where notice that C_{k^0} ribosomes are bound to the RBSs of the k-th protein-coding gene transcripts and $r_t^{p_k} = E_{mk}(l_{pk}, l_e) C_{k^0} = J_k(\mu, r) r$ are actively involved in translation.

An analogous expression can be obtained for the number r_r of ribosomes bound to complexes involved in translation of ribosomes themselves:

$$r_b^r = N_r \left(C_{r^0} + \sum_{j=1}^{n_r} C_{r^j} \right) = N_r \left[1 + \frac{1}{E_{mr}(l_{p_r}, l_e)}\right] J_r(\mu, r) r \tag{26}$$

where we have taken into account that it requires N_r protein species to build-up a ribosome and we consider an average ribosomal protein.

Therefore, the number of mature available ribosomes r_a can be obtained from:

$$\begin{aligned} r_a &= r + N_r \left(C_{r^0} + \sum_{j=1}^{n_r} C_{r^j} \right) + \sum_{k=1}^{N_{nr}} \left[C_{k^0} + \sum_{j=1}^{n_r} C_{k^j} \right] + \sum_{k=1}^{N_{\text{exo}}} \left[C_{k^0} + \sum_{j=1}^{n_r} C_{k^j} \right] \\ &= r + N_r \left[1 + \frac{1}{E_{mr}(l_{p_r}, l_e)}\right] J_r(\mu, r) r + \sum_{k=1}^{N_{nr}+N_{\text{exo}}} \left[1 + \frac{1}{E_{mk}(l_{pk}, l_e)}\right] J_k(\mu, r) r \end{aligned} \tag{27}$$

where N_{nr} is the number endogenous non-ribosomal host protein-coding genes and N_{exo} that of exogenous ones.

Notice for a ribosomal density $l_e = 25$ and average ribosomal and non-ribosomal protein lengths (see Table SI.2) the average values $1/E_{mr} = 0.21$ and $1/E_{mp} = 0.12$ are small, accounting for the percentage of ribosomes bound to the RBS.

From equation (27) we get the number of free ribosomes r as a function of the mature available ones r_a:

$$r = \frac{r_a}{1 + N_r \left[1 + \frac{1}{E_{mr}(l_{p_r}, l_e)}\right] J_r(\mu, r) + \sum_{k=1}^{N_{nr}+N_{\text{exo}}} \left[1 + \frac{1}{E_{mk}(l_{pk}, l_e)}\right] J_k(\mu, r)} \tag{28}$$

SI.4. Fractions of bound and actively translating ribosomes with respect to the available mature ones

Notice from (25) and (26) that the number of bound and bound-actively-translating ribosomes is:

$$\begin{aligned} r_b^r &= N_r \left[1 + \frac{1}{E_{mr}(l_{p_r}, l_e)}\right] J_r(\mu, r) r \ , & r_t^r &= N_r J_r(\mu, r) r \\ r_b^{nr} &= \sum_{k=1}^{N_{nr}} \left[1 + \frac{1}{E_{mk}(l_{pk}, l_e)}\right] J_k(\mu, r) r \ , & r_t^{nr} &= \sum_{k=1}^{N_{nr}} J_k(\mu, r) r \\ r_b^{\text{exo}} &= \sum_{k=1}^{N_{\text{exo}}} \left[1 + \frac{1}{E_{mk}(l_{pk}, l_e)}\right] J_k(\mu, r) r \ , & r_t^{\text{exo}} &= \sum_{k=1}^{N_{\text{exo}}} J_k(\mu, r) r \end{aligned} \tag{29}$$

Using (28) and:

$$\text{WSum}(\mu, r) \triangleq N_r \left[1 + \frac{1}{E_{mr}(l_{p_r}, l_e)}\right] J_r(\mu, r) + \sum_{k=1}^{N_{nr}+N_{\text{exo}}} \left[1 + \frac{1}{E_{mk}(l_{pk}, l_e)}\right] J_k(\mu, r) \tag{30}$$

we can define the fractions of ribosomes bound to complexes and those actively involved in translation relative to the mature available ones for ribosomal, non-ribosomal and exogenous protein-coding genes:

$$
\begin{aligned}
\Phi_t^r &\triangleq \frac{N_r J_r(\mu,r)}{1+\mathrm{WSum}(\mu,r)} = \frac{r_t^r}{r_a}\ , &\quad \Phi_b^r &\triangleq \frac{N_r\left[1+\frac{1}{E_{mr}(l_{p_r},l_e)}\right] J_r(\mu,r)}{1+\mathrm{WSum}(\mu,r)} = \frac{r_b^r}{r_a} \\
\Phi_t^{nr} &\triangleq \frac{\sum_{j=N_{nr}} J_j(\mu,r)}{1+\mathrm{WSum}(\mu,r)} = \frac{r_t^{nr}}{r_a}\ , &\quad \Phi_b^{nr} &\triangleq \frac{\sum_{k=N_{nr}}\left[1+\frac{1}{E_{mk}(l_{pk},l_e)}\right] J_k(\mu,r)}{1+\mathrm{WSum}(\mu,r)} = \frac{r_b^{nr}}{r_a} \\
\Phi_t^{exo} &\triangleq \frac{\sum_{j=\mathrm{exo}} J_j(\mu,r)}{1+\mathrm{WSum}(\mu,r)} = \frac{r_t^{nr}}{r_a}\ , &\quad \Phi_b^{exo} &\triangleq \frac{\sum_{k=N_{\mathrm{exo}}}\left[1+\frac{1}{E_{mk}(l_{pk},l_e)}\right] J_k(\mu,r)}{1+\mathrm{WSum}(\mu,r)} = \frac{r_b^{nr}}{r_a}
\end{aligned}
\tag{31}
$$

where $\sum_{k=N_{nr}}, \sum_{k=N_{\mathrm{exo}}}$ stand for the sum over the ensemble of all genes coding for endogenous and exogenous non-ribosomal proteins respectively.

Thus, for the native host cell we can consider:

$$
\Phi_t^h \triangleq \Phi_t^r + \Phi_t^{nr}\ ,\ \ \Phi_b^h \triangleq \Phi_b^r + \Phi_b^{nr} \tag{32}
$$

and the analogous $\Phi_t^s = \Phi_t^h + \Phi_t^{exo}$ and $\Phi_b^s = \Phi_b^h + \Phi_b^{exo}$ for the strain hosting exogenous protein-coding genes.

Using (30) and (31), the fraction of ribosomes bound to translating complexes (including both translating complexes for endogenous and exogenous proteins and those bound to RBSs) relative to the available mature ribosomes is:

$$
\Phi_b = \frac{\mathrm{WSum}(\mu,r)}{1+\mathrm{WSum}(\mu,r)} \tag{33}
$$

Notice each term

$$
\frac{\left[1+\frac{1}{E_{mj}}\right] J_j(\mu,r)}{1+\sum_{k=\{r,nr,exo\}}\left[1+\frac{1}{E_{mk}}\right] J_k(\mu,r)}
$$

can be understood as the share of cell resources required to express the $j-$th protein-coding gene. Thus, the magnitude of the adimensional coefficient $J_j(\mu,r)$ is a measure of the resources recruited to express the $j-$th protein-coding gene.

SI.5. Obtaining the cell specific growth rate

Cell growth can essentially be explained as the time variation of the protein fraction of the total cell mass. Yet, not all protein mass contributes to cell growth. On the one hand, there are proteins which may be undergoing active degradation. On the other, exogenous proteins will in general not have any active role in the cell that contributes to its growth. Therefore, next we consider only the endogenous ribosomal and non-ribosomal proteins to obtain the cell specific growth rate from the time variation of the endogenous protein fraction of the total cell mass.

To deal with protein degradation, we take into account that the protein fraction of cell mass is the sum of the mass of functional and non-functional proteins (ie. proteins undergoing degradation). Though non-functional proteins do not contribute to cell growth, they do to cell mass. Thus, for a k-th protein species we can consider the fraction quantity of functional molecules of the protein, p_k, and the one of non-functional ones p_k^{nf} so that the total number of protein copies of the k-th species is $p_k^T = p_k + p_k^{nf}$. Then, considering the dynamics (19) of a generic protein, we have:

$$
\begin{aligned}
\dot{p}_k &= \frac{\nu_t(s_i)}{l_{pk}} J_k(\mu,r) r - (d_k+\mu) p_k \\
\dot{p}_k^{nf} &= d_k p_k - \mu p_k^{nf}
\end{aligned}
\tag{34}
$$

where we have taken into account that the non-functional fraction p_k^{nf} only undergoes dilution due to cell growth and there is a conversion from functional to non-functional fraction caused by protein degradation.

If we consider the average mass of an amino acid m_{aa}, the mass weight of a protein of length l_{pk} can be approximated as $m_{aa}l_{pk}$. Thus, for p_k^T molecules of the k-th protein, their total mass weight is $m_k = m_{aa}l_{pk}p_k^T$. Then, the mass of the N_{nr} non-ribosomal endogenous proteins in the cell proteins can be approximated as:

$$m_{nr} = \sum_{k=1}^{N_{nr}} m_{aa}l_{pk}\left(p_k + p_k^{nf}\right) \tag{35}$$

Therefore, the times variation of the protein mass explained by this set of N_{nr} proteins is:

$$\begin{aligned}
\dot{m}_{nr} &= \sum_{k=1}^{N_{nr}} m_{aa}l_{pk}\left(\dot{p}_k + \dot{p}_k^{nf}\right) \\
&= m_{aa}\sum_{k=1}^{N_{nr}} l_{pk}\left[\frac{\nu_t(s_i)}{l_{pk}}J_k(\mu,r)r - (d_k+\mu)p_k\right] + m_{aa}\sum_{k=1}^{N_{nr}} l_{pk}\left(d_k p_k - \mu p_k^{nf}\right) \\
&= m_{aa}\frac{\nu s_i}{K_{sc}+s_i}\sum_{k=1}^{N_{nr}} J_k(\mu,r)r - \mu m_{nr} \\
&= m_{aa}\nu_t(s_i)\Phi_t^{nr}r_a - \mu m_{nr}
\end{aligned} \tag{36}$$

where recall $J_k(\mu,r)r$ is the number of ribosomes bound to complexes involved in the translation of each k-th protein and we have used the definitions (31) in the last step. Notice, in addition, that the degradation of proteins does not play a role when we consider the dynamics of the protein mass. It indeed plays a role when we consider the number of active proteins.

As for the protein cell mass variation explained by the time variation in the total number of ribosomes r_T we will have the analogous expression:

$$\dot{m}_{r_T} = m_{aa}\nu_t(s_i)\Phi_t^r r_a - \mu m_{r_T} \tag{37}$$

where we have used the fact that N_r ribosomal proteins are required to form up one ribosome. Notice that we only consider the weight of the protein fraction of the ribosomes. This accounts only for approximately one third of the ribosomes mass [15].

Denoting the *host* cell protein weight $m_h = m_{nr} + m_{r_T}$, we reach the expression:

$$\dot{m}_h = m_{aa}\nu_t(s_i)\left(\Phi_t^r + \Phi_t^{nr}\right)r_a - \mu m_h \tag{38}$$

which, using (32), can be expressed as:

$$\dot{m}_h = m_{aa}\nu_t(s_i)\Phi_t^h r_a - \mu m_h \tag{39}$$

Recall that the specific growth rate μ is a continuous approximation of the discrete event process of cell duplication. Here we consider that the total biomass dry weight variation (ie. that of the whole population of cells) is mainly caused by cell duplication (i.e. population growth), and the dynamics of cell mass accumulation are much faster than those of cell duplication. Under this assumption, we may consider the protein mass for each cell quickly reaches steady state ($\dot{m}_h \approx 0$). Thus, from equation (39) we get the expression for the cell specific growth rate:

$$\mu = \frac{m_{aa}}{m_h}\nu_t(s_i)\Phi_t^h r_a \tag{40}$$

Notice $\Phi_t^h r_a$ is the number of ribosomes actively translating endogenous proteins (both ribosomal and non-ribosomal) at a given time instant. Equation (40) allows to predict this number given a specific growth rate, assuming saturation of intracellular substrate (eg. considering a batch experiment and the exponential growth phase) and considering the average values for the amino acids mass and that of the protein fraction of the cell.

Notice also that (40) can be expressed as a function of the total number of ribosomes r_T as:

$$\mu = \frac{m_{aa}}{m_h}\nu_t(s_i)\Phi_t^h\Phi_m r_T \tag{41}$$

SI.6. Cell specific growth rate and population dynamics

Our model considers the intracellular substrate s_i as source of building blocks to synthesize proteins. Using the model in a multi-scale framework considering the macroscopic dynamics of the population of cells and the uptake of extracellular substrate requires relating the cell population growth rate with the individual cell one. To this end, we next relate the expression for the cell specific growth rate μ obtained in (40) with the classical Monod-like expressions for the growth of a population of cells.

Recall if we have a population of N cells and we consider the average cell dry mass m_{cDW}, the total biomass dry-weight will be $M_b = N m_{cDW}$. By taking derivative with respect to time, we get:

$$\dot{M}_b = \dot{N} m_{cDW} + N \dot{m}_{cDW} \tag{42}$$

Now, consider the continuous approximation of cell duplication:

$$\dot{N} = \mu N \tag{43}$$

where $\mu = \log 2/t_d$, with t_d the cell population doubling time, is the specific growth rate. Then:

$$\dot{M}_b = \mu M_b + N \dot{m}_{cDW} \tag{44}$$

from which we get:

$$\dot{m}_{cDW} = \frac{\dot{M}_b}{N} - \mu m_{cDW} \tag{45}$$

where $\frac{\dot{M}_b}{N}$ is the mean value per cell of the population mass growth.

As done in section SI.5, we assume the cell mass quickly reaches steady state as compared to the population dynamics. Thus, from equation (45) and assuming $\dot{m}_{cDW} \approx 0$ we get:

$$\mu = \frac{\dot{M}_b}{N m_{cDW}} = \frac{\dot{M}_b}{M_b} \tag{46}$$

Experimental evidence suggests that the cell densityρ varies little throughout the adult cell life [10], so:

$$\mu = \frac{\dot{M}_b}{M_b} = \frac{\rho \dot{M}_b}{\rho M_b} = \frac{\dot{V}_b}{V_b} \tag{47}$$

The specific growth rate under a limiting substrate obtained from the experimental macroscopic analysis of a culture of cells can be expressed using the classical empirical Monod relationship:

$$\mu(s) = \frac{\dot{M}_b}{M_b} = \frac{\mu_m s}{K_s + s} \tag{48}$$

where μ_m is the maximum specific growth rate, K_s is the Monod affinity constant, and s is the concentration of the limiting substrate in the culture medium.

The identities (47) allow to relate the specific growth rate obtained in (40) with the one obtained from population-scale macroscopic experiments under the condition of steady-state growth. Under this condition, the rate of total cell-mass growth is identical to the rate of cell number growth [27]. Thus:

$$\mu = \frac{\mu_m s}{K_s + s} = \frac{m_{aa}}{m_h} \frac{\nu s_i}{K_{sc} + s_i} \Phi_t^h r_a \tag{49}$$

To relate the intracellular and extracellular substrate, we resorted to the theoretical approaches to derive the Monod equation. Several alternatives exist [13, 26, 24]. We followed a reasoning derived from the model developed in [25], where the quantity of intracellular substrate s_i is related to the one of extracellular substrate s through the dynamics of nutrient import and catabolism:

$$\dot{s}_i = e_t \frac{\nu_t s}{\frac{k_t}{V_m} + s} - e_m \frac{\nu_m s_i}{k_m + s_i} - \mu s_i \tag{50}$$

where V_m is the harvest volume [24], e_t and e_m are transport and catabolism enzymes, and Michaelis-Menten kinetics are assumed (see [25]).

If we assume that nutrient import quickly balances nutrient catabolism and we neglect the dilution term:

$$\dot{s}_i \approx 0 \rightsquigarrow s_i = \frac{\frac{k_m}{c-1} s}{\frac{k_t}{V_m}\frac{c}{c-1} + s} \tag{51}$$

where $c = \frac{e_m \nu_m}{e_t \nu_t}$. Notice if the maximum import and catabolism fluxes are balanced, ie. $c \approx 1$, then there is a linear relationship between the intracellular amount of substrate and its extracellular concentration. Otherwise, if catabolism is more efficient than transport ($c > 1$) the intracellular amount of substrate s_i saturates with increasing values of s.

Recall in our model the specific growth rate (49) is a function of s_i. Using (51) we obtain the Monod-like expression:

$$\frac{s_i}{K_{sc} + s_i} = \frac{\frac{k_m}{K_{sc}(c-1)+k_m} s}{\frac{K_{sc} k_t c}{V_m[K_{sc}(c-1)+k_m]} + s} \tag{52}$$

If we assume that the Michaelis-Menten constant for substrate catabolism is the same as the constant we defined in (3), that is, $K_{sc} = k_m$, we have:

$$\frac{s_i}{K_{sc} + s_i} = \frac{\frac{1}{c} s}{\frac{k_t}{V_m} + s} \tag{53}$$

Notice that the hypothesis $K_{sc} = k_m$ implicitly implies that the Michaelis-Menten constants for substrate catabolism and transport have similar values ($k_t = k_m$), in agreement with the assumptions in [25].

Also notice the term $1/c = \frac{e_t \nu_t}{e_m \nu_m}$ can be interpreted as the maximum flux yield between nutrient import and its catabolism. If $c \approx 1$, that is, under the hypothesis that the efficiency of nutrient import and catabolism are balanced so the maximum import and catabolism fluxes are similar:

$$\frac{s_i}{K_{sc} + s_i} = \frac{s}{\frac{k_t}{V_m} + s} \tag{54}$$

In case catabolism is more efficient than transport ($c > 1$) we will need an increase in the concentration of the substrate in the extracellular medium (s) to achieve the same value of $\frac{s_i}{K_{sc}+s_i}$ in (53) as compared to the balanced case $c = 1$. Finally, in case transport is more efficient than catabolism ($c < 1$) we will need lower concentrations of the extracellular substrate.

Then, from (49) ,(48) and (53) we get:

$$\mu(s) = \frac{\frac{1}{c}\frac{m_{aa}}{m_h}\nu \Phi_t^h r_a s}{\frac{k_t}{V_m} + s} = \frac{\mu_m s}{K_s + s} \tag{55}$$

so we can identify:

$$\begin{aligned} \mu_m &= \frac{1}{c}\frac{m_{aa}}{m_h}\nu \Phi_t^h r_a \\ K_s &= \frac{k_t}{V_m} \end{aligned} \tag{56}$$

Note: The relationship (53) is valid but in the extreme cases non relevant cases $c = 0$ (there is transport into the cell but nutrients are not metabolised) and $c = \infty$. In these cases dilution cannot be neglected in equation (50) and the equilibria are different.

SI.7. Relationship between growth rate and cell mass

Our model accounts for the protein mass distribution but does not obtain the total protein cell mass. A simple approach to solve this would be considering a constant average cell protein mass. Yet, the cell mass varies with growth rate. In this section we consider the relationship between growth rate and the total cell protein mass, and obtain an empirical model relating the host protein mass m_h with the specific growth rate μ.

Several phenomenological models have been proposed in the literature accounting for the relationship between growth rate and cell dry weight, like the recent ones [20, 27]. In [27] a Monod-like relation between the chromosome replication-segregation period $C + D$ and the cell specific growth rate μ is considered:

$$C + D = \frac{\alpha + \beta\mu}{\mu} \tag{57}$$

We estimated the parameters α and β in (57) using the data in [3]. Figure SI.3(left) shows the good fit obtained.

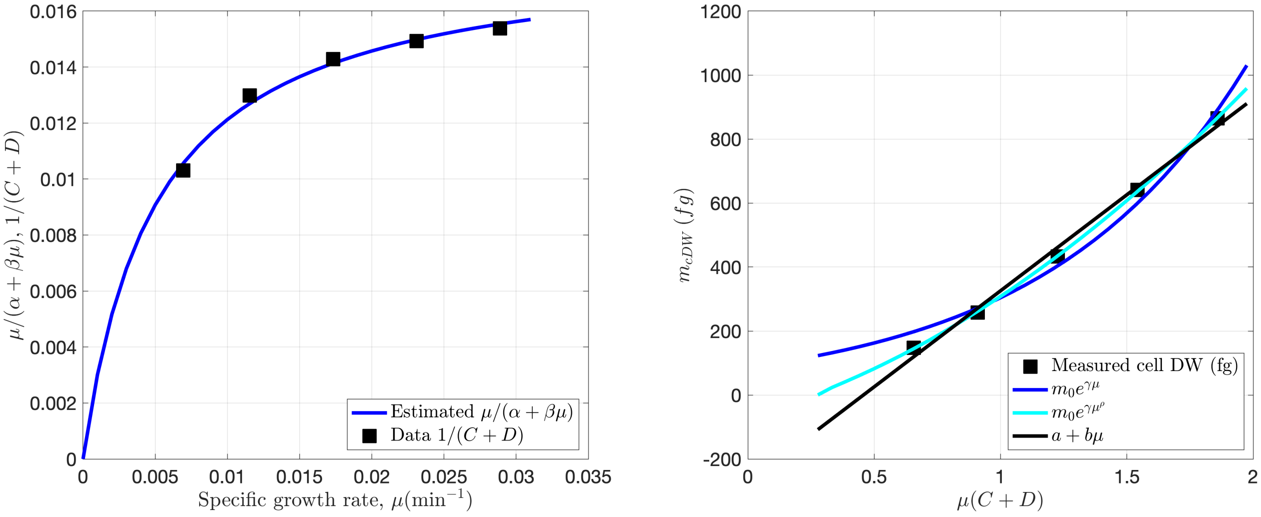

Figure SI.3. (Left:) Best fitting results for the identity (57) using the data in [3]. (Right:) Alternative fittings between the cell dry weight m_c and the product $\mu\,(C + D)$.

In addition the authors in [27] propose a linear relation between the cell dry weight and the product of $C + D$ and μ:

$$m_c = m_0\mu(C + D) \tag{58}$$

Therefore, according to this model, there is an affine relationship between the cell dry weight and specific growth rate:

$$m_c = m_0(\alpha + \beta\mu) \tag{59}$$

As shown in Figure SI.3(right), for the data we used, the relationship (58) gives a very rough approximation. Indeed, as shown in the same figure, and affine relationship gives much better fit.

As an alternative, in [20] an exponential relationship is proposed between the cell volume S_c and the product of $\mu(C + D)$:

$$S_c = S_0 e^{\mu(C+D)} \tag{60}$$

Assuming constant cell density, equation (60) can be expressed as a function of the cell dry weight. Figure SI.3(right) shows the fit assuming constant cell density, so that equation (60) can be expressed as a function of the cell dry weight. Notice that a better fit can be obtained considering the modified relationship:

$$m_c = m_0 e^{\gamma\mu(C+D)} \tag{61}$$

Now, if we consider expressions (57) and (61), we get:

$$m_c = m_0 e^{\gamma(\alpha+\beta\mu)} = \bar{m}_0 e^{\bar{\gamma}\mu} \tag{62}$$

where $\bar{m}_0 = m_0 e^{\gamma\alpha}$ and $\bar{\gamma} = \gamma\beta$.

Notice equation (62) is the solution of the differential equation:

$$\frac{dm_c}{d\mu} = \bar{\gamma} m_c \tag{63}$$

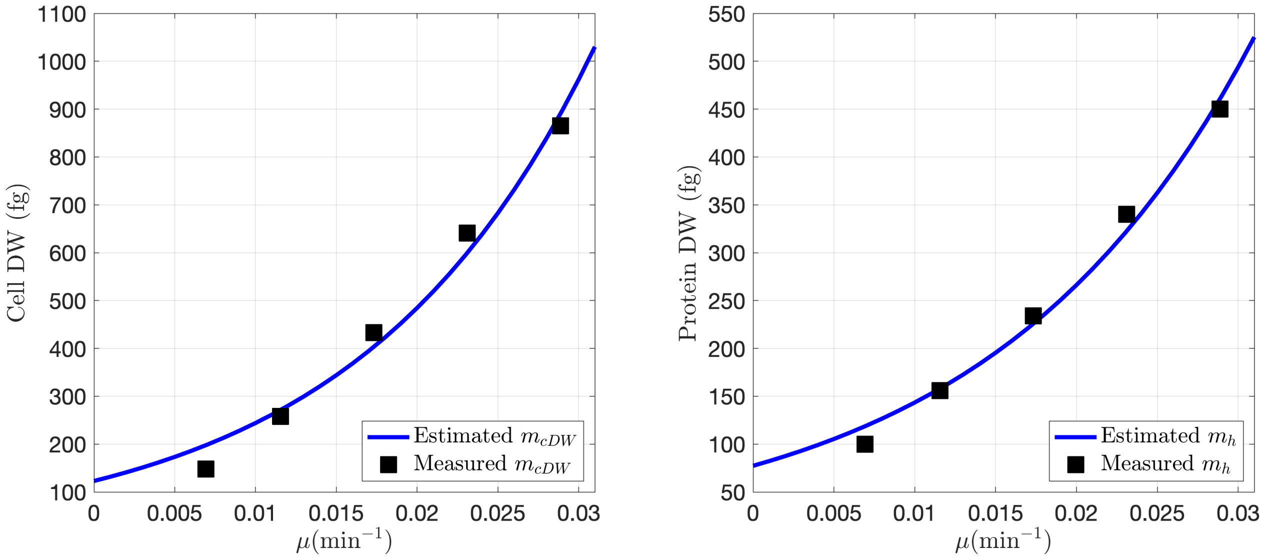

Figure SI.4. Fit between the cell dry weight (left) and the protein content dry weight (right) as a function of the specific growth rate using the expressions (63) and (64) respectively.

**Model**	**Model parameters**	
$m_c = \bar{m}_{c0} e^{\bar{\gamma}\mu}$	$\bar{m}_{c0} = 123.022\, 10^{-15}$ (g)	$\bar{\gamma} = 68.5547$ (min)
$m_h = \bar{m}_{h0} e^{\beta\mu}$	$\bar{m}_{h0} = 77.3748e\, 10^{-15}$ (g)	$\beta = 61.7813$ (min)

Table SI.1. Phenomenological models relating cell and protein dry mass and specific growth rate and their best fit parameters for the data in [3].

which expresses that the variation of cell mass (dry weight) relative to the variation of the specific growth rate is proportional to the cell mass. Figure SI.4 (left) shows the results obtained for (62) and Table SI.1 lists the best fitted parameters. Better fits can be obtained with alternative phenomenological expressions to (62) at the cost of losing the simple interpretation provided by expression (63).

For the relationship between the cell content of endogenous proteins and the specific growth rate we postulate a relationship analogous to (63). Thus, we consider:

$$\frac{dm_h}{d\mu} = \beta m_h \tag{64}$$

Figure SI.4 (right) shows the results obtained and Table SI.1 lists the parameters corresponding to the best fit.

Notice that alternative phenomenological relationships can be used instead, with possibly better fit to the experimental data. Thus, for instance, the power law model $m_h = \bar{m}_0 e^{\bar{\gamma}\mu^{\nu}}$ with $\bar{m}_0 = 1.2918\, 10^{-14}$, $\bar{\gamma} = 14.1089$ and $\nu = 0.389$ is an alternative.

SI.8. Average host dynamics and steady state balanced growth.

We were interested in having an average model for the host dynamics and its steady state that can be used as base for analyzing host-circuit interactions. To this end we considered, on the one hand, the dynamics (37) of the ribosomal protein mass content of host, m_{r_T} and, on the other, the dynamics (36) of the mass of the ensemble of non-ribosomal endogenous proteins m_{nr}.

For the non-ribosomal endogenous proteins we consider them as a lumped species with a single average

resources recruitment strength $J_{nr}(\mu, r)$ and average E_{mnr} such that:

$$N_{nr}\left[1+\frac{1}{E_{mnr}}\right]J_{nr}(\mu,r)=\sum_{k=1}^{N_{nr}}\left[1+\frac{1}{E_{mk}(l_{pk},l_e)}\right]J_k(\mu,r) \tag{65}$$

We also considered average values for the endogenous protein lengths l_p^r and l_p^{nr} and a common average amino acid mass m_{aa} so that the masses are related to the number of proteins as:

$$m_{r_T} = m_{aa}N_r l_p^r r_T = m_{rib} r_T \tag{66}$$

$$m_{nr} = m_{aa}N_{nr} l_p^{nr} p_{nr} \tag{67}$$

where m_{rib} is the average mass of the protein content of ribosomes and p_{nr} includes both functional and inactive endogenous proteins (see SI.5).

Then, using (37), (36), the expression for the growth (40) and the definitions (31), the dynamics for the host endogenous ribosomal and non-ribosomal protein mass can be expressed as:

$$\dot{m}_{r_T} = \mu\left[m_h(\mu)\frac{N_r J_r(\mu,r)}{N_r J_r(\mu,r)+N_{nr}J_{nr}(\mu,r)} - m_{r_T}\right] \quad ; \quad J_r(\mu,r) = 0.62\frac{l_p^r}{l_e}\omega_r\frac{1}{\frac{d_m^r}{K_{C^0}^r(s_i)}+\mu r} \tag{68}$$

$$\dot{m}_{nr} = \mu\left[m_h(\mu)\frac{N_{nr} J_{nr}(\mu,r)}{N_r J_r(\mu,r)+N_{nr}J_{nr}(\mu,r)} - m_{nr}\right] \quad ; \quad J_{nr}(\mu,r) = 0.62\frac{l_p^{nr}}{l_e}\omega_{nr}\frac{1}{\frac{d_m^{nr}}{K_{C^0}^{nr}(s_i)}+\mu r} \tag{69}$$

with the average effective RBS strengths

$$K_{C^0}^k(s_i) = \frac{K_b^k}{K_u^k + \frac{\nu_t(s_i)}{l_e}} \tag{70}$$

for $k = \{r, nr\}$.

The number of free ribosomes is obtained using the averaged (28):

$$r = \frac{\Phi_m r_T}{1+\left(1+\frac{1}{E_{mr}}\right)N_r J_r(\mu,r)+\left(1+\frac{1}{E_{mnr}}\right)N_{nr}J_{nr}(\mu,r)} \tag{71}$$

with r_T obtained from (66), and the specific growth rate is obtained from (40) using the fraction

$$\Phi_t^h = \frac{N_r J_r(\mu,r)+N_{nr}J_{nr}}{1+\left(1+\frac{1}{E_{mr}}\right)N_r J_r(\mu,r)+\left(1+\frac{1}{E_{mnr}}\right)N_{nr}J_{nr}(\mu,r)} \tag{72}$$

Notice from (68)–(69) that the steady state will reached either when the growth rate stalls ($\mu = 0$) or when there exists exponential balanced growth with:

$$\frac{m_{r_t}}{m_h(\mu)} = \frac{N_r J_r(\mu,r)}{N_r J_r(\mu,r)+N_{nr}J_{nr}(\mu,r)} \tag{73}$$

$$\frac{m_{nr}}{m_h(\mu)} = \frac{N_{nr} J_{nr}(\mu,r)}{N_r J_r(\mu,r)+N_{nr}J_{nr}(\mu,r)} \tag{74}$$

and the growth rate (41):

$$\mu = \frac{m_{aa}}{m_p}\nu_t(s_i)\Phi_t^h\Phi_m r_T$$

Thus, at steady state balanced growth the relative resources recruitment strength provides the relative mass fractions at steady state. It is easy to see that this holds also for individual proteins. The relative resources recruitment strength of a given protein equals its relative mass in the cell.

Notice from (68), (71) and (41) that at steady state:

$$\mu_{ss}(s_i) = \frac{m_{aa}}{m_{rib}}\nu_t(s_i)\Phi_m\Phi_t^r \tag{75}$$

that is, the growth rate at steady state depends linearly on the fraction $\Phi_m \Phi_t^r$ of bound ribosomes actively being used to build up ribosomes (ie. actively translating the transcripts) relative to the total number of ribosomes.

At steady state, the flux of free resources for a given intracellular substrate s_i, defined as the number of free ribosomes times the cell growth rate, can be obtained as:

$$\mu_{ss} r_{ss}(s_i) = m_{aa} \nu_t(s_i) \left(\frac{\Phi_m \Phi_t^r}{m_{rib}} \right)^2 \frac{1 - \Phi_b^s}{\Phi_t^h} m_h(\mu_{ss}) \tag{76}$$

showing a linear relationship with the cell protein weight m_p.

The host cell protein weight m_h depends of the growth rate. To calculate it, we used both the data available in [3] and the phenomenological relationships obtained in section SI.7.

SI.9. Synthesis rate of exogenous proteins and interaction with the host cell

Product titer and productivity rate are important measures of performance in biotechnological applications. In this section we consider the expression of an exogenous protein-coding gene and we analyze how the number of produced proteins at steady state and its specific mass productivity rate (synthesis rate) vary as a function of its RBS and promoter strengths and the interaction with the host cell. The expressions obtained can easily be adapted for the analysis of the synthesis rate of an endogenous protein as a function of its RBS and promoter strengths and interaction with the remaining host endogenous genes.

We define the synthesis rate of a protein A, Π_{nA}, as the number molecules of A at steady-state balanced growth produced per cell and generation as in [12]. Notice this is equivalent to the productivity rate of the protein A. Thus, if we consider a population of N cells and the continuous approximation of cell duplication (43), the total quantity of molecules of the protein A, P_A^N, will increase as the population of cells does as:

$$\dot{P}_A^N = p_{A,\text{ss}} \dot{N} = p_{A,\text{ss}} \mu N \tag{77}$$

where $p_{A,\text{ss}}$ is the number of molecules of the protein A at steady state in a single cell. Therefore:

$$\Pi_{nA} = \frac{\dot{P}_A^N}{N} = p_{A,\text{ss}} \mu \tag{78}$$

Analogously, we can defined the mass synthesis rate as:

$$\Pi_A = m_{A,\text{ss}} \mu \tag{79}$$

For the host endogenous dynamics we considered the average model as described in section SI.8. We extend the model by adding the dynamics of the exogenous protein of interest A as:

$$\dot{m}_A = \mu \left[m_h(\mu) \frac{N_A J_A(\mu, r)}{N_r J_r(\mu, r) + N_{nr} J_{nr}(\mu, r)} - m_A \right] \quad ; \quad J_A(\mu, r) = 0.62 \frac{l_p^A}{l_e} \omega_A \frac{1}{\frac{d_m^A}{K_{C^0}^A} + \mu r} \tag{80}$$

where notice the denominator in the fraction of RRSs only includes the host protein-coding genes and the mass $m_h(\mu)$ is still that of the native host cell and not the mass of the strain $m_s = m_h(\mu) + m_A$.

The expressions (68)-(69) for the host endogenous dynamics remain the same. Yet, now the number of free ribosomes r is obtained using (28):

$$r = \frac{\Phi_m r_T}{1 + \text{WSum}(\mu, r)}$$

with

$$\text{WSum}(\mu, r) = \left(1 + \frac{1}{E_{mr}}\right) N_r J_r(\mu, r) + \left(1 + \frac{1}{E_{mnr}}\right) N_{nr} J_{nr}(\mu, r) + \left(1 + \frac{1}{E_{mA}}\right) N_A J_A(\mu, r)$$

and the specific growth rate is obtained from (40) using the fraction

$$\Phi_t^h = \frac{N_r J_r(\mu, r) + N_{nr} J_{nr}(\mu, r)}{1 + \text{WSum}(\mu, r)} \tag{81}$$

The exogenous proteins do not contribute to cell growth, but contribute to cell mass. Thus, the fraction of fraction ϕ_A of synthesized exogenous protein(s) must take into account their mass contribution. To this end, we take into account the specific growth rate is obtained from (40):

$$\mu = \frac{m_{aa}}{m_h} \nu_t(s_i) \Phi_t^h r_a$$

and the mass dynamics for the exogenous protein:

$$\dot{m}_A = m_{aa} \nu_t(s_i) \Phi_t^{nr} r_a - \mu m_A \tag{82}$$

From (82) evaluated at steady state, we get

$$\mu = \frac{m_{aa}}{m_A} \nu_t(s_i) \Phi_t^A r_a$$

Therefore, we obtain the relationship:

$$\frac{m_A}{m_h} = \frac{\Phi_t^A}{\Phi_t^h} = \frac{N_A J_A(\mu, r)}{N_r J_r(\mu, r) + N_{nr} J_{nr}(\mu, r)} \tag{83}$$

Now, the protein mass content of the strain is

$$m_s(\mu) = m_h(\mu) + m_A(\mu) = \left(1 + \frac{\Phi_t^A}{\Phi_t^h}\right) m_h(\mu) = \frac{\Phi_t^s}{\Phi_t^h} m_h(\mu) \tag{84}$$

Therefore, the mass fraction of protein A is:

$$\phi_A(\mu) \triangleq \frac{m_A(\mu)}{m_s(\mu)} = \frac{\Phi_t^A}{\Phi_t^s} = \frac{N_A J_A(\mu, r)}{N_r J_r(\mu, r) + N_{nr} J_{nr}(\mu, r) + N_A J_A(\mu, r)} \tag{85}$$

Using (80) evaluated at steady state and the results above, the synthesis rate (79) becomes:

$$\Pi_A = m_{A,\text{ss}} \mu = m_h(\mu) \frac{N_A J_A(\mu, r)}{N_r J_r(\mu, r) + N_{nr} J_{nr}(\mu, r)} \mu = m_s(\mu) \frac{N_A J_A(\mu, r)}{N_r J_r(\mu, r) + N_{nr} J_{nr}(\mu, r) + N_A J_A(\mu, r)} \mu \tag{86}$$

SI.10. Model parameters

Table SI.2 shows the set of parameters used in the model.

SI.11. Estimation of the fractions Φ_t^h and Φ_b^h

Next, for the host native *E. coli* cell, we evaluate the fractions Φ_b^h and Φ_t^h of bound ribosomes and bound ribosomes being actively used in translating complexes relative to the mature available ones as a function of the number of free ribosomes r (see the definitions (32) in Section SI.4). We expect a very low number of free ribosomes. If this was not the case, there would be no real competition to recruit them. To set an initial upper limit, we use the estimation $r = \mathbf{N}(350, 35)$ in [11]. In addition, having too many free ribosomes in excess would imply a superfluous us of energy for the cell. Considering this hypothesis, we evaluated equation (33) as a function of the mature available ribosomes r_a and the free ones r. Figure SI.5(left) shows the values estimated. Notice the values of Φ_b^h close to $\Phi_b^h = 1$ indicating that the cell is always at the edge of its maximum capacity for using the available resources.

From the estimation of the fraction Φ_b^h and using the definitions in section SI.4 we can obtain that of the dimensionless sum $\sum_{k=\{r,nr\}} \left[1 + \frac{1}{E_{mk}}\right] J_k(\mu, r)$ reflecting the resources recruitment load generated by the

Model parameters for ***E. coli***				
**Parameter**	**Description**	**Value**	**Unit**	**Reference**
t_d	duplication time	$[20, 60]$	min	[15]
μ	specific growth rate	$[0.01, 0.035]$	min^{-1}	$\mu = \log 2/t_d$
$y_{x/s}$	yield biomass on glucose	0.5	gDW/g glucose	
$\bar{l}_p$	average non-ribosomal protein length	333	amino acids (aa molec^{-1})	calculated from data in [9]
$\bar{l}_r$	average ribosomal protein length	195	amino acids (aa molec^{-1})	calculated from data in [9]
l_e	ribosome occupancy length	$[20, 30]$	aa molec^{-1}	estimated ([21, 7, 6, 16])
$l_{e,\text{min}}$	ribosome occlusion length	11	aa molec^{-1}	[19]
ν	maximum translation speed per ribosome	1260	$\text{aa} \cdot \text{min}^{-1}$	[15, 18]
k_u^k	dissociation rate constant RBS-ribosome	[6, 135]	min^{-1}	[5, 11]
k_b^k	association rate constant RBS-ribosome	[3, 15]	$\text{molec}^{-1} \cdot \text{min}^{-1}$	[5, 17, 11]
$t_{\frac{1}{2},\text{mRNA}}$	average mRNA half-life	$[2, 6]$	min	[1, 4, 9, 8]
$d_{m,\text{ss}}$	median mRNA degradation rate constant (steady phase)	0.17	min^{-1}	[1]
d_m	median mRNA degradation rate constant (exponential growth)	0.28	min^{-1}	[4]
η	maximum transcription speed	$[1500, 3000]$	$\text{aa} \cdot \text{min}^{-1}$	[15, 14]
m_{aa}	average amino acid mass	110	Da	[22]
m_{aa}	average amino acid mass	$182.6\,10^{-24}$	g	[22]
m_p	geometric average of cell protein weight	$224\,10^{-15}$	gDW	[15, 3]

Table SI.2. Model parameters.

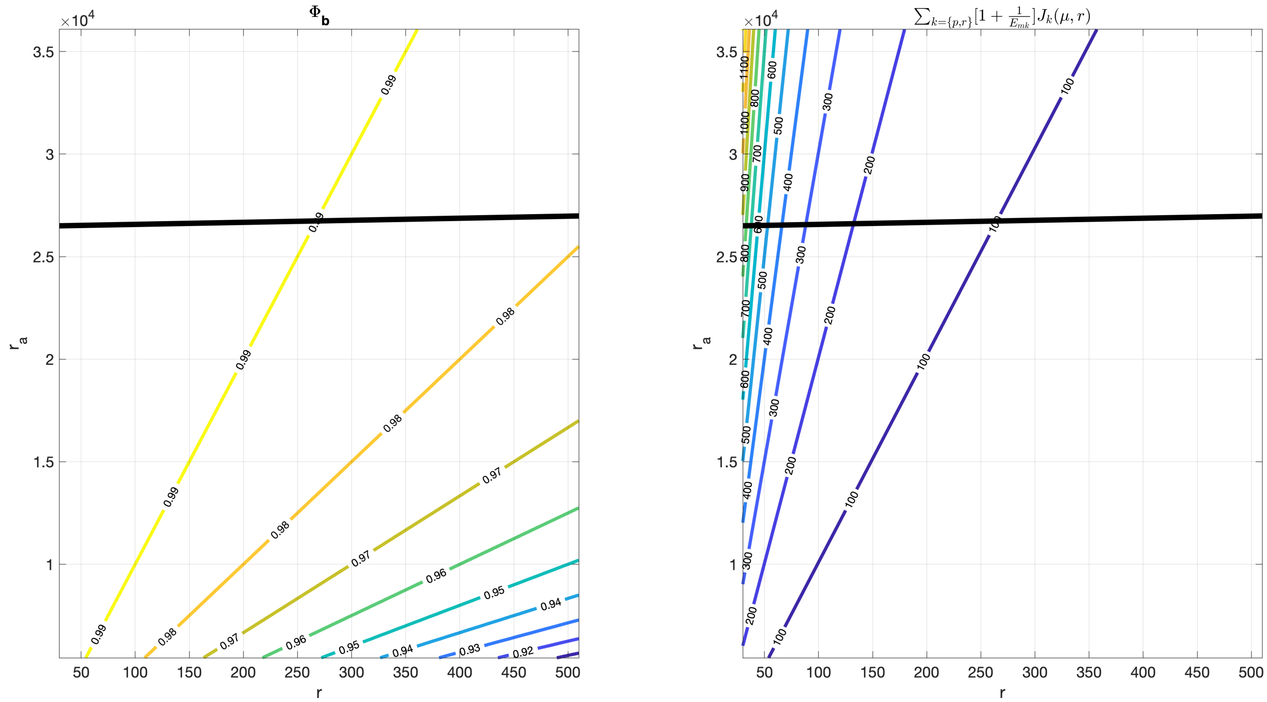

Figure SI.5. (Left:) Fraction Φ_b^h of ribosomes bound to translating complexes relative to the mature available ones r_a as a function of r_a and the number of free ribosomes r. (Right:) Corresponding estimation of the weighted sum $\sum_{k=\{r,nr\}}\left[1+\frac{1}{E_{mk}}\right]J_k(\mu,r)$. The black line in both subplots depicts the value of mature available ribosomes r_a as a function of the free ones r using the number of bound ribosomes obtained from the data in [9].

whole set of ribosomal and non-ribosomal endogenous proteins being expressed at a given moment in the cell (see Figure SI.5(right)).

To validate the estimations above, we evaluated both the sum $\sum_{k=\{r,nr\}} J_k(\mu,r)$ and the weighted sum $\sum_{k=\{r,nr\}}\left[1+\frac{1}{E_{mk}}\right]J_k(\mu,r)$ for all protein-coding genes reported in [9] for *E. coli*.

The dynamic model for the expression of protein p in [9] considers:

$$\dot{p} = \frac{\beta_p\beta_m}{d_m} - \mu p \tag{87}$$

where β_m (mRNA/t) is the transcription rate, β_p (protein/(mRNA·t)) the translation one and d_m the mRNA degradation rate constant. For those transcripts without information for d_m in [9] we used the value shown in Table SI.2. Then, we used the equation (19) to derive the relationship:

$$J_k = \frac{l_{pk}}{\nu_t(s_i)}\frac{\beta_p\beta_m}{d_m}\frac{1}{r} \tag{88}$$

We took into account that the data was obtained for fast growing cells, with doubling time $t_d = 21$ minutes. Under this condition, it is sensible to consider that the intracellular substrate will be saturated and the cells are growing at its maximum growth rate., so that $\nu_t(s_i) = \nu$. We evaluated (88) considering the set of all non-ribosomal proteins, the ribosomal ones, and the full set of proteins, and obtained the corresponding fractions Φ_t^h and Φ_b^h.

Figure SI.6(left) shows the experimental values of Φ_t^h and Φ_b^h obtained as a function of the number of free ribosomes r. As expected, and in agreement with the estimations shown in Figure SI.5(left), the values of Φ_b^h kept very close to 1 for a wide range of values of the number of free ribosomes. Recall the data in [9] was obtained for fast growing cells for which it is sensible to consider saturated intracellular substrate so that

the cells are growing at its maximum growth rate and the number of free ribosomes is very small. This is consistent with the result shown in Figure SI.5(left, black line) where the estimated values of Φ_b are plotted along with the value of mature available ribosomes r_a as a function of the free ones r using the number of bound ribosomes obtained from the data in [9]. In order to keep the experimental values of Φ_b^h over 0.98, the number of free ribosomes must keep below the limit of a few hundreds. Notice also the experimental constant ratio $\Phi_t^h/\Phi_b^h \approx 0.83$. in agreement with [2, 3]. That is, around 17% of the total number of mature available ribosomes are, in average, located at the RBSs.

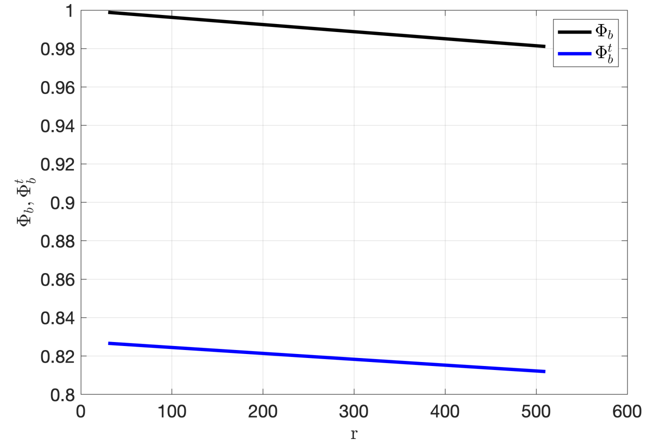

Figure SI.6. Experimental values of Φ_b^h (black) and Φ_t^h (blue) as a function of the number of free ribosomes r obtained using the data in [9]. The ratio $\Phi_t^h/\Phi_b^h = 0.828$ is constant.

SI.12. Evaluation of the maximum resources recruitment strength.

In this section we evaluate, on the one hand, the order of magnitude of the resources recruitment strength for the protein-coding genes in *E. coli*. This is useful to evaluate the maximum burden (measured as the sum of the RRSs) in the native*E. coli* host cell and estimate how many genes are active.

We used the data in [9] and expression (88) to calculate the individual values of maximum resources recruitment strength J_k evaluated at $r = 1$ for the set of non-ribosomal and ribosomal protein-coding genes and sorted them by magnitude and as the ratio between the sorted values of J_k and the corresponding protein lengths. The results are shown in Figure SI.7.

As a proxy to estimate how many genes are active at a given time we calculated the cumulative sum of the maximum resources recruitment strengths and obtained how many genes being expressed are required to explain both 95% and 99% of the total cumulative sum. We did this independently for both ribosomal and non-ribosomal proteins. Figure SI.8 shows the results obtained. Notice the same results will be obtained if using the weighted resources recruitment strengths since the ratio Φ_t^h/Φ_b^h is constant.

Our results show that out of the 68 ribosomal genes, 49 of them (72%) explain 95% of the cumulative sum of the maximum resources recruitment strength of the ribosomal genes. To explain 99% we need 57 ribosomal genes (84% of them). On the other hand, for non-ribosomal genes we need 875 out of 3551 genes (25%) to explain 95% of the cumulative sum and 1735 (49%) to explain the 99%.

SI.13. Estimation of the number of free ribosomes in the cell

Estimation of the number of free ribosomes in the cell, r, is key for assessing the competition among the cell circuits for cellular resources. The results in the previous sections suggest that an extremely low number of free ribosomes, with order of magnitude in the range 10^1, gives a high sensitivity of the total cumulative sum of the maximum resources recruitment strength with respect to variations in the amount of free ribosomes (see Figures SI.5 and SI.6) while an order of magnitude in the range 10^2 is enough to keep a robust value of

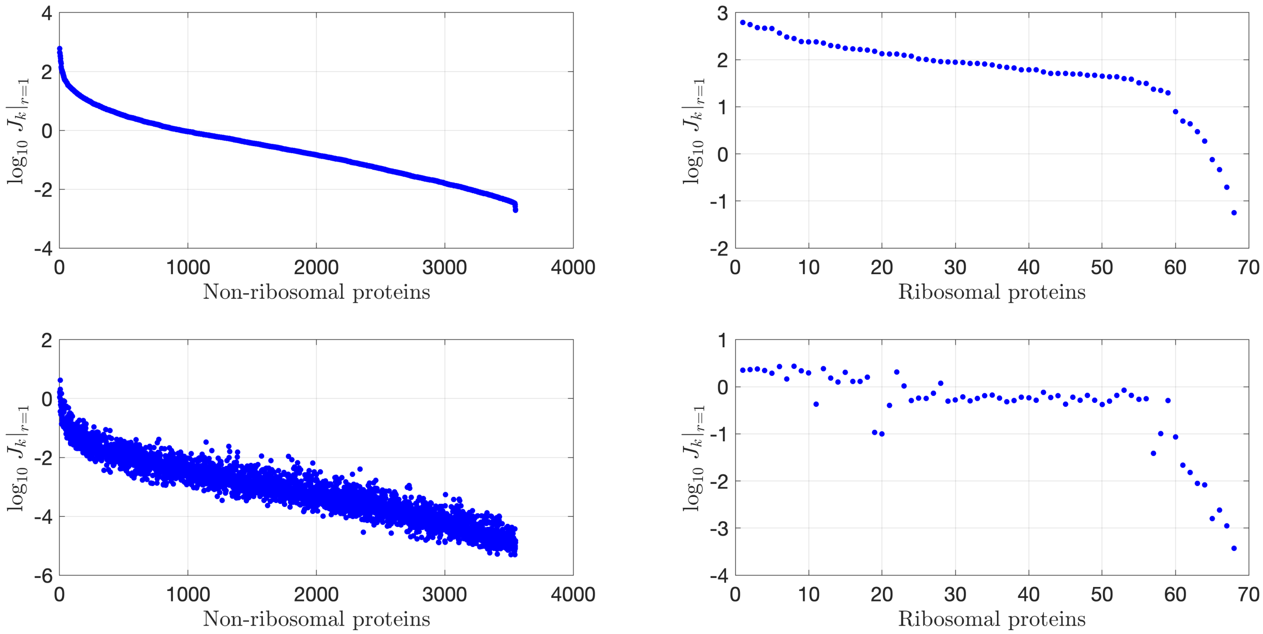

Figure SI.7. (Top:) Maximum resources recruitment strength $J_k(r = 1)$ for the set of non-ribosomal (left) and ribosomal protein-coding genes (right) in *E. coli* sorted by magnitude in logarithmic scale. (Bottom:) Ratio between the sorted maximum resources recruitment strength $J_k(r = 1)$ and the corresponding protein length (aa).

the total amount of recruited resources with respect to variations in the number of free ribosomes. That is, by not expressing superfluous resources, the cell forces a competition for them that induces a high sensitivity of the total amount of recruited resources with respect to variations in the number of free ribosomes, while a small surplus of superfluous resources induces robustness.

To evaluate the range of expected values of r we used experimental data of the translation efficiency per mRNA. Notice from the dynamics (19) for a protein p_k we can define:

$$Y_{p/\text{mRNA}} \triangleq \frac{\nu}{l_{pk}} \frac{J_k r}{\omega_k} = 0.62 \frac{\nu}{l_e} \frac{r}{\frac{d_{mk}}{K_{C0}^k(s_i)} + \mu r} \frac{s_i}{K_{sc} + s_i} \tag{89}$$

where s_i is the copy number of molecules of intracellular substrate, d_{mk} is the mRNA degradation rate constant, recall $K_{C0}^k(s_i)$ is a substrate dependent parameter essentially related to the RBS strength and we consider that substrate availability will only affect translation and not transcription (see Section SI.1). Notice $Y_{p/\text{mRNA}}$ is the number of protein copies produced per transcript.

We used the data from [9] to estimate an upper bound for the number of free ribosomes r using (89) and the values for $Y_{p/\text{mRNA}}$ obtained using (87):

$$Y_{p/\text{mRNA}} = \frac{\beta_{pk}}{d_{mk}} \tag{90}$$

Then, the relationship between the RBS strength-related term K_{C0}^k and the free ribosomes r becomes:

$$0.62 \frac{\nu}{l_e} \frac{r}{\frac{d_{mk}}{K_{C0}^k(s_i)} + \mu_m f(s_i) r} f(s_i) = \frac{\beta_{pk}}{d_{mk}} \tag{91}$$

where $f(s_i) = s_i/(K_{sc} + s_i)$ and we have used equations (54), (55) and (56) relating the specific growth rate μ with the maximum one μ_m and the availability of intracellular substrate. Notice that, for any given protein and intracellular substrate availability, the number of free ribosomes will determine the required value of the

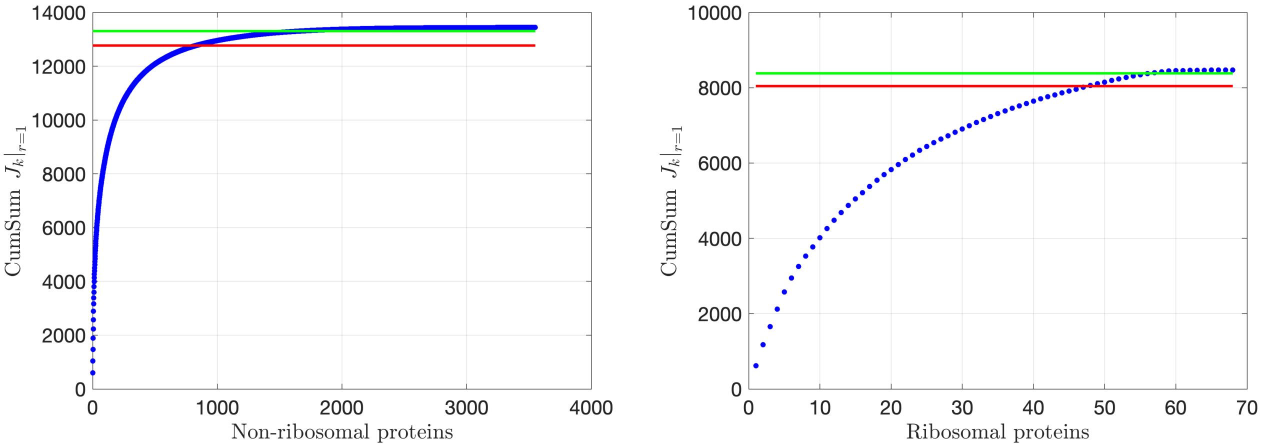

Figure SI.8. Cumulative maximum resources recruitment strength for the set of non-ribosomal (left) and ribosomal protein-coding genes (right) in *E. coli.* The horizontal lines correspond to the 95% (red) and 99% (green) levels respectively.

RBS strength-related term $K_{C^0}^k(s_i)$ to attain the experimental value of the translation efficiency per mRNA $Y_{p/\mathrm{mRNA}}$.

The translation efficiency given by expression (91) depends on the ribosomes density $1/l_e$. An average ribosomes density around 4.2 ribosomes per 100 codons in optimal growth conditions has been reported in the literature for the prokaryote *L. lactis* [16]. This value is the same we obtained from the data in (88) by considering the total number of available active ribosomes $\Phi_b r_a$ obtained in Section SI.12 (see Figure SI.6) and dividing it by the sum of the lengths of all proteins weighted by a factor 0.5 to account for the estimation that 50% of the genes are active (explain 99% of the cumulative sum of the maximum resources recruitment strength). Similar values are found for other organisms [7]. For *E. coli* a value of 3.5 is given in [15]. The ribosomes density is inversely log-linearly related to the length of the coding sequence, with a slope quite consistent for a variety of organisms [7]. To account for this, we approximated a power law consistent with the findings in [7] and resulting in 4 ribosomes per 100 codons for an average protein length of 330 codons. We obtained the relationship:

$$\frac{1}{l_e} = \frac{0.0703}{l_{pk}^{0.097}} \tag{92}$$

This gives a range $l_e \subset [18,\, 31]$, with a value $l_e = 25$ for the average protein length. The minimum value is consistent with the shortest protein length (18 codons) in the database we used.

Figure SI.9 shows the results obtained for the set of all ribosomal and non-ribosomal proteins and their average values.

From the results shown in Figure SI.9, notice that the number of free ribosomes r required to explain the experimental value of the translation efficiency per mRNA $Y_{p/\mathrm{mRNA}}$ for each protein increases as the value of the RBS strength-related term $K_{C^0}^k$ decreases. Indeed, the more free ribosomes are available, the less competition for shared resources. The number of free ribosomes r is an indicator of the level of competition for resources. Thus, expression (91) implies that a gene producing short-living transcripts will require, for the same level of competition, a stronger RBS to achieve the same translation efficiency per mRNA $Y_{p/\mathrm{mRNA}}$ as one with long-living transcripts.

To estimate an upper limit for the copy number of free ribosomes required to achieve the experimental translation rates per mRNA, we considered an upper bound for the RBS strength-related term $K_{C^0}^k$.

Recall from equation (10) that $K_{C^0}^k$ term is a function of the intracellular substrate availability. Since the data we used from [9] was obtained for fast growing cells, we can consider intracellular substrate saturation. Under this condition we get:

$$K_{C^0}^k = \frac{K_b^k}{K_u^k + \frac{\nu}{l_e}} \tag{93}$$

Using the value of ν in Table SI.2 and the range of l_e given above, we estimate $\frac{\nu}{l_e} \subset [40,\, 70]$ $(\mathrm{molec}^{-1} \cdot \mathrm{min}^{-1})$.

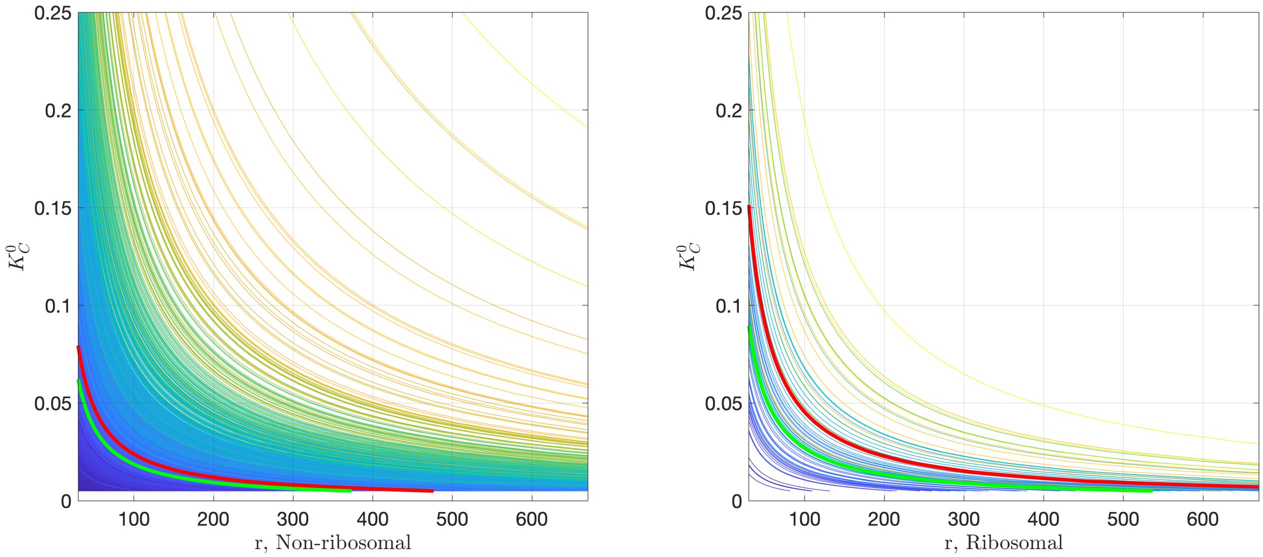

Figure SI.9. Relationship between the RBS strength-related term $K_{C^0}^k$ and the number of free ribosomes r obtained using the experimental data in [9] for non-ribosomal (left) and ribosomal (right) proteins in *E. coli*. Thin lines correspond to the experimental value of the translation efficiency per mRNA $Y_{p/\text{mRNA}}$ for each protein. The red thick line corresponds to the mean for all proteins in the corresponding non-ribosomal and ribosomal sets. The green thick line corresponds to the approximated mean when the term associated to the maximum specific cell growth rate is neglected in the expression (91).

On the other hand, the values of the association and dissociation rates of the ribosome to the RBS, K_b^k and K_u^k, may vary in a large range. Values $K_b^k \subset [3,\ 15]$ (molec$^{-1} \cdot$ min^{-1}) are found in the literature (see Table SI.2). We use a conservative upper bound $K_b^{\max} = 10$ (molec^{-1}) considering binding is diffusion controlled. From the literature, we consider a range for the dissociation rate $K_u^k \subset [3,\ 135]$ (min^{-1}). Overall, these estimates give us a range (under the assumption of intracellular substrate saturation) $K_{C^0}^k \subset [0.02,\ 0.2]$ (molec^{-1}).

From the results shown in Figure SI.9, notice that a maximum number of free ribosomes $r \approx 350$ can confidently explain the translation efficiencies per mRNA $Y_{p/\text{mRNA}}$ for almost all proteins while maintaining the value of the RBS strength-related term $K_{C^0}^k < 0.2$. This estimation for the amount of free ribosomes is in complete agreement with the estimation $r = \mathbf{N}(350, 35)$ in [11].

With the upper limit $r \approx 350$ we could explain the translation efficiencies per mRNA $Y_{p/\text{mRNA}}$ calculated as $\frac{\beta_{pk}}{d_{mk}}$ (see equation (91)) using the data in [9] but for a small set of 80 non-ribosomal proteins out of 3551 (2.25%). In 52 of them, this could be attributed to their extremely long-living transcripts. In the remaining 28 ones, to their very high translation efficiency per mRNA $Y_{p/\text{mRNA}}$ expected from their values of β_{pk} and d_{mk} given in [9]. This can be explained by rewriting (91) as:

$$Y_{p/\text{mRNA}} = 0.62 \frac{\nu}{l_e} \frac{f(s_i) \frac{K_{C^0}^k(s_i)}{d_{mk}} r}{1 + \mu_m f(s_i) \frac{K_{C^0}^k(s_i)}{d_{mk}} r} = \frac{\beta_{pk}}{d_{mk}} \tag{94}$$

Figure SI.10 shows a plot of the function (94), as a function of its argument $f(s_i) K_{C^0}(s_i) r / d_m$ for two mRNA degradation rates corresponding to short and long-living mRNAs and ribosomes densities in the range $l_e = [18, 31]$. Notice $Y_{p/\text{mRNA}} = \frac{\beta_{pk}}{d_{mk}}$ saturates at the maximum attainable value:

$$Y_{p/\text{mRNA},\max} = 0.62 \frac{\nu}{l_e} \frac{1}{\mu_m} \tag{95}$$

Saturation is reached for lower values of the argument $f(s_i) K_{C^0}(s_i) r / d_m$ as the transcripts have longer half-lives, i.e. smaller values of the degradation rate d_m.

The few cases our model could not predict all have values of $\frac{\beta_{pk}}{d_{mk}}$ above $Y_{p/\text{mRNA},\max}$ for the values of ν and l_e used in the model.

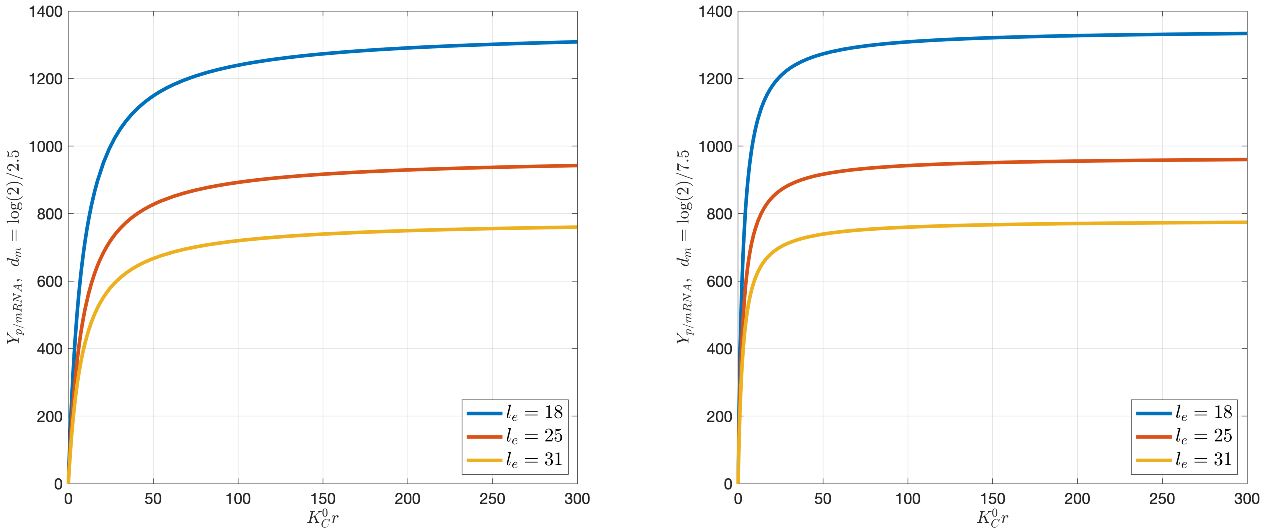

Figure SI.10. Translation efficiency per mRNA $Y_{p/\mathrm{mRNA}}$ as a function of $f(s_i)K_{C^0}r$ for two mRNA degradation rates corresponding to mRNA half-lives 2.5 minutes (left) and 7.5 minutes (right) for ribosomes densities corresponding to $l_e = \{18, 25, 31\}$ codons.

On the other hand, it is interesting to notice that for very low values of $f(s_i)K_{C^0}(s_i)r/d_m$ such that $f(s_i)K_{C^0}(s_i)r \ll \frac{d_m}{\mu_m}$ we can approximate:

$$Y_{p/\mathrm{mRNA}} \approx 0.62\frac{\nu}{l_e}f(s_i)\frac{K^k_{C^0}(s_i)}{d_{mk}}r = \frac{\beta_{pk}}{d_{mk}} \tag{96}$$

from which we get:

$$\beta_{pk} \approx 0.62\frac{\nu}{l_e}f(s_i)K^k_{C^0}(s_i)r \tag{97}$$

That is, for a highly competitive scenario where the number of free ribosomes is sufficiently small (e.g. in the order of few tens to few hundreds for typical values of d_{mk}, $\mu_m = 0.032\,\mathrm{min}^{-1}$ and $f(s_i)K_{C^0}(s_i)$ at its maximum estimated value $f(s_i)K_{C^0}(s_i) = 0.2$) the translation rate (proteins per mRNA per time unit) is proportional to the ribosomes density $\frac{0.62}{l_e}$, the effective maximum translation rate per codon attainable for a given substrate availability $\nu f(s_i)$, the RBS strength $K^k_{C^0}$, and the available free ribosomes r.

Notice under this scenario the translation rate will suffer large stochastic fluctuations caused by stochastic fluctuations in the number of free ribosomes. In this case, the transcription rate for a given RBS-strength mainly depends on the competition for cellular resources and, therefore, on the number of free ribosomes r, and it is largely independent of the specific growth rate.

SI.14. Estimated fractions of ribosomes

Additional results for Section 2.2 are given here. The total number of ribosomes (both experimental and estimated) much increases for very fast growing cells. Thus, the fraction of free ribosomes with respect to the total number only increases from 0.08% up to 1.37% for cell duplication times between 100 and 24 minutes respectively even though the number of free ribosomes multiplies by almost 200-fold (see Figure SI.11). The estimated value of the fraction of mature ribosomes with respect to the total number of ribosomes was $\Phi_m \approx 0.90$ and the estimated fraction of active bound ribosomes kept roughly constant with growth rate at $\Phi^h_t\Phi_m \approx 0.78$.

Notice also the logarithmic affine relationship between the number of free ribosomes r and its flux μr, ($\log_{10}(r) \approx 4.07 + 0.78\log_{10}(\mu r)$) reflecting a power-law relationship between growth rate and number of free ribosomes.

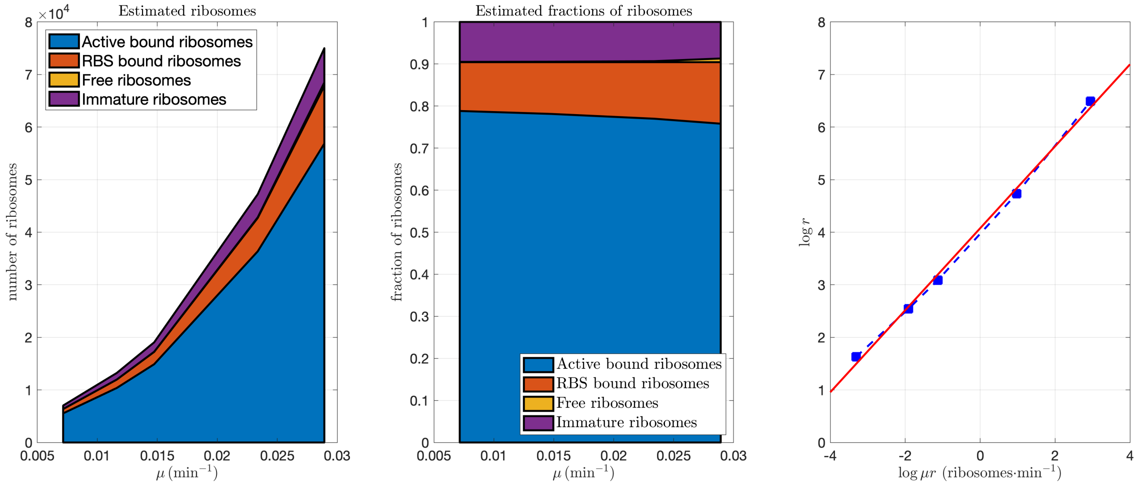

Figure SI.11. Estimated number of ribosomes (Left) fractions of ribosomes (Center) and logarithmic affine relationship between the number of free ribosomes r and its flux μr (Right)

SI.15. Effect of substrate availability of growth rate and specific synthesis rate

Additional images SI.12 and SI.13 for Results Section 2.4 are given here. In both cases, the effect of substrate variation is considered. The function $f(s_i)$ corresponds to the normalized attainable peptide synthesis rate defined in equation (4), so that:

$$f(s_i) \triangleq \frac{\nu_t(s_i)}{\nu} = \frac{s_i}{K_{sc} + s_i} \tag{98}$$

Therefore $f(s_i)$ is a saturated monotonous increasing function with the intracellular substrate s_i, taking values in the range $[0, 1]$.

SI.16. Software code and data

The software code and data are available at `https://github.com/sb2cl/Resources-allocation-NoPi21`

References

[1] Jonathan A. Bernstein, Arkady B. Khodursky, Pei-Hsun Lin, Sue Lin-Chao, and Stanley N. Cohen. Global analysis of mrna decay and abundance in escherichia coli at single-gene resolution using two-color fluorescent dna microarrays. *Proceedings of the National Academy of Sciences*, 99(15):9697–9702, 2002.

[2] Evert Bosdriesz, Douwe Molenaar, Bas Teusink, and Frank J. Bruggeman. How fast-growing bacteria robustly tune their ribosome concentration to approximate growth-rate maximization. *The FEBS Journal*, 282(10):2029–2044, 2015.

[3] H. Bremer and P. P. Dennis. Modulation of chemical composition and other parameters of the cell by growth rate. *EcoSal Plus*, 2008.

[4] Huiyi Chen, Katsuyuki Shiroguchi, Hao Ge, and Xiaoliang Sunney Xie. Genome-wide study of mRNA degradation and transcript elongation in E scherichia coli . *Molecular Systems Biology*, 11(1):781, 2015.

[5] Maarten H. de Smit and Jan van Duin. Translational standby sites: How ribosomes may deal with the rapid folding kinetics of mrna. *Journal of Molecular Biology*, 331(4):737 – 743, 2003.

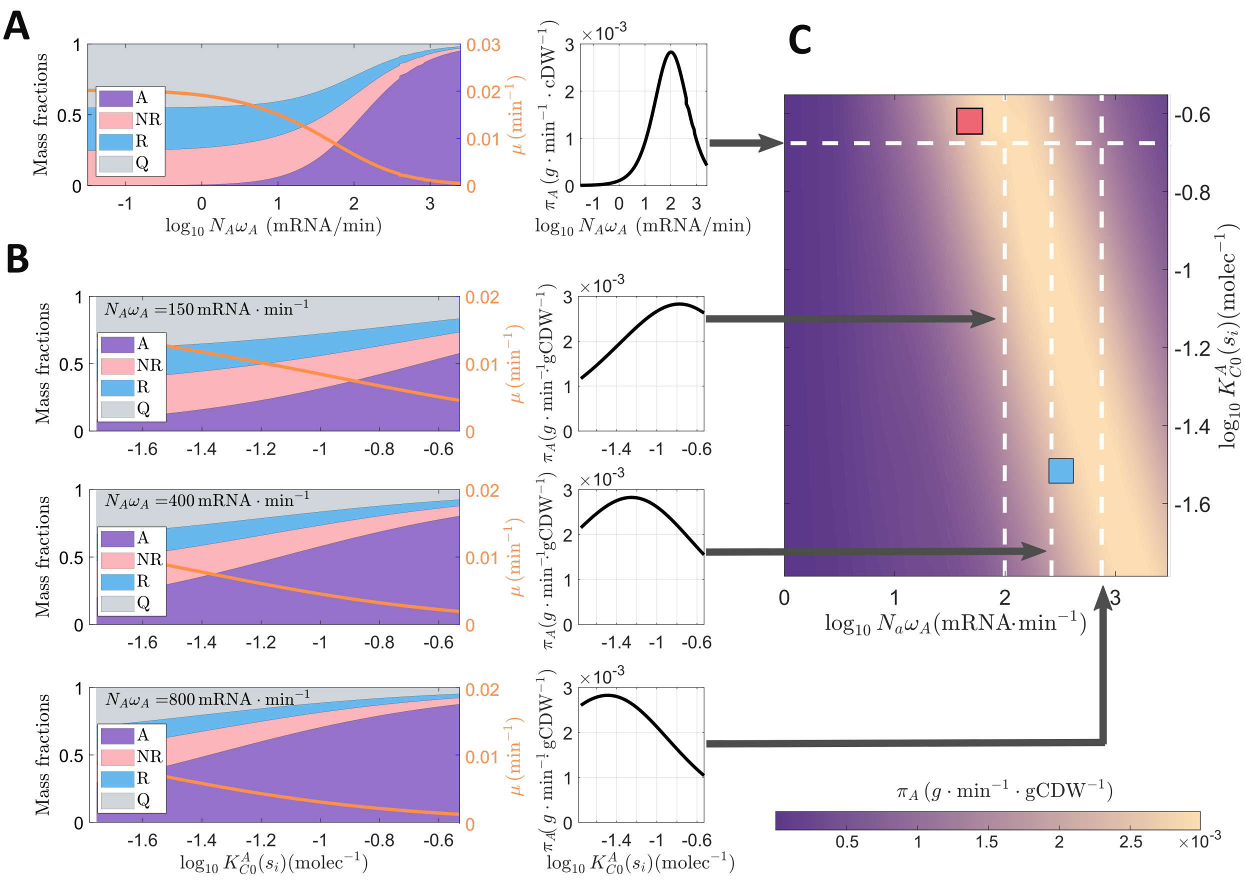

Figure SI.12. **A:** Effect of increasing mRNA synthesis rate on growth rate and mass fractions (*left*) and specific protein synthesis rate (*right*). **B:** Effect of RBS strength variation on growth rate and mass fractions (*left*) and specific protein synthesis rate (*right*) for the three values $N_A\omega_A = \{150, 400, 800\}$. **C:** Specific protein synthesis rate across the expression space $N_A\omega_A, K^A_{C0}$. The pink and blue squares correspond to the average lumped values of $N_x\omega_x, K^x_{C0}(s_i)$ for the non-ribosomal (pink) and ribosomal (blue) endogenous protein coding-genes in an *E. coli* host respectively. The dashed white lines correspond to the four scenarios analyzed in the panels A and B.

[6] Mette Eriksen, Kim Sneppen, Steen Pedersen, and Namiko Mitarai. Occlusion of the ribosome binding site connects the translational initiation frequency, mrna stability and premature transcription termination. *Frontiers in Microbiology*, 8:362, 2017.

[7] L.D. Fernandes, A.P.S.d. Moura, and L. Ciandrini. Gene length as a regulator for ribosome recruitment and protein synthesis: theoretical insights. *Sci Rep*, 7:17409, 2017.

[8] Peter Großmann, Anja Lück, and Christoph Kaleta. Model-based genome-wide determination of rna chain elongation rates in escherichia coli. *Scientific Reports*, 7:17213, 2017.

[9] Jean Hausser, Avi Mayo, Leeat Keren, and Uri Alon. Central dogma rates and the trade-off between precision and economy in gene expression. *Nature Communications*, 10(1):68, 2019.

[10] Kubitschek HE., Baldwin WW, Schroeter SJ., and Graetzer R. Independence of buoyant cell density and growth rate in escherichia coli. *J Bacteriol.*, 158(1):296–299, 1984.

[11] Andrzej M. Kierzek, Jolanta Zaim, and Piotr Zielenkiewicz. The Effect of Transcription and Translation Initiation Frequencies on the Stochastic Fluctuations in Prokaryotic Gene Expression. *Journal of Biological Chemistry*, 276(11):8165–8172, 2001.

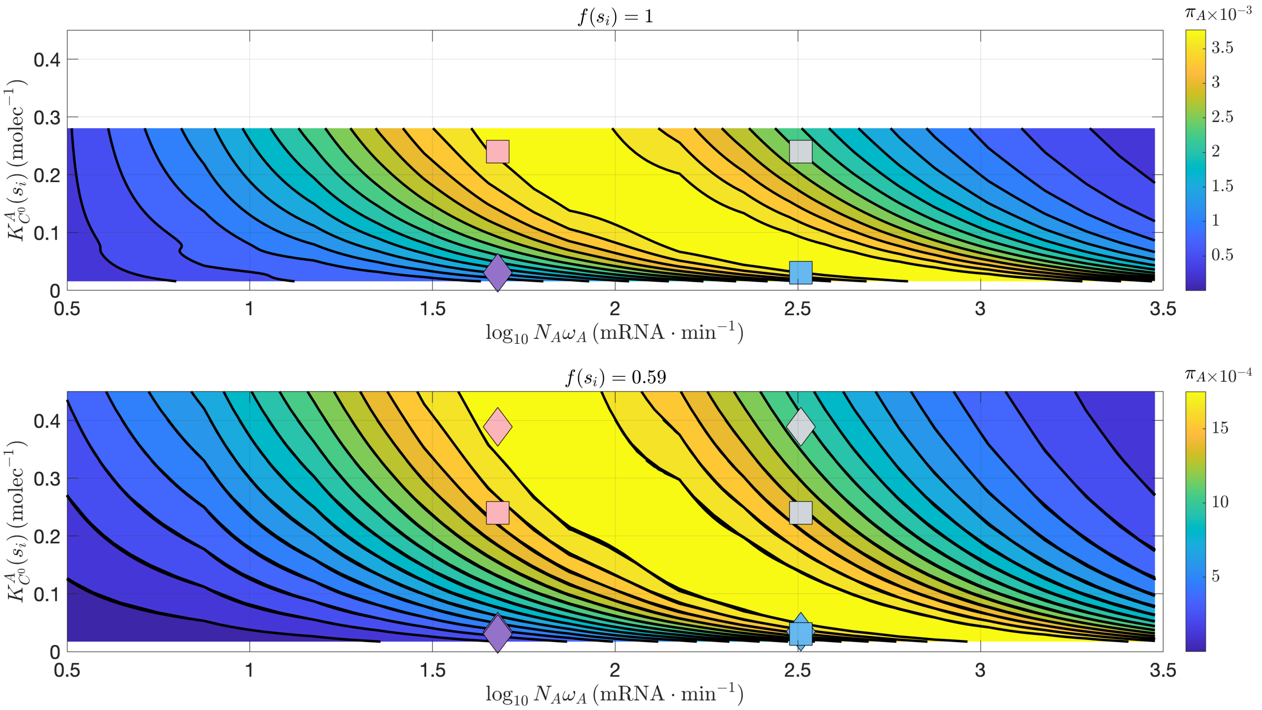

Figure SI.13. Effect of the variation of substrate on the specific optimal synthesis rate of exogenous protein-coding genes. The contour lines show the specific synthesis rate as a function of the effective RBS strength and the mRNA synthesis rate. They were obtained by simulating the expression of a generic exogenous protein-coding gene with varying strengths across the expression space $N_A\omega_A, K^A_{C^0}$. We considered genes with four differential characteristics. **Top:** Saturated substrate. **Bottom:** Low substrate condition. The values of RBS strength and mRNA synthesis rate are now depicted as diamonds for the new scenario. The values of the previous substrate-saturated scenario are kept as squares for the sake of comparison. Recall the dependence of the effective RBS strength $K^x_{C^0}(s_i)$ on the availability (tantamount, nutrient quality) of the intracellular substrate. In our model, strong RBSs are more affected by variations of the substrate than weak ones. As the substrate decreases, the effective RBS-strength for genes with weak RBSs do not appreciably change. Notice that these genes require less resources (per gene) than the ones with strong RBSs. For the genes with strong RBSs, the apparent RBS strength increases as the substrate decreases. They increase their avidity for scarce resources. This way, they both keep their relative positions within the specific synthesis rate space..

[12] Gene Wei Li, David Burkhardt, Carol Gross, and Jonathan S. Weissman. Quantifying absolute protein synthesis rates reveals principles underlying allocation of cellular resources. *Cell*, 157(3):624–635, 2014.

[13] Yu Liu. Overview of some theoretical approaches for derivation of the monod equation. *Appl Microbiol Biotechnol*, 73:1241–1250, 2007.

[14] Jason Lloyd-Price, Sofia Startceva, Vinodh Kandavalli, Jerome G. Chandraseelan, Nadia Goncalves, Samuel M. D. Oliveira, Antti H´akkinen, and Andre S. Ribeiro. Dissecting the stochastic transcription initiation process in live escherichia coli. *DNA Research*, 23(3):203–214, 03 2016.

[15] Ron Milo, Paul Jorgensen, Uri Moran, Griffin Weber, and Michael Springer. Bionumbers–the database of key numbers in molecular and cell biology. *Nucleic acids research*, 38(Database issue):D750–3, January 2010.

[16] F. Picard, P. Loubire, and L. et al. Girbal. The significance of translation regulation in the stress response. *BMC Genomics*, 14(588), 2013.

[17] Howard M. Salis. Chapter two - the ribosome binding site calculator. In Christopher Voigt, editor, *Synthetic Biology, Part B*, volume 498 of *Methods in Enzymology*, pages 19 – 42. Academic Press, 2011.

[18] Matthew Scott, Carl W. Gunderson, Eduard M. Mateescu, Zhongge Zhang, and Terence Hwa. Interdependence of cell growth and gene expression: Origins and consequences. *Science*, 330(6007):1099–1102, 2010.

[19] Gilad Shaham and Tamir Tuller. Genome scale analysis of escherichia coli with a comprehensive prokaryotic sequence-based biophysical model of translation initiation and elongation. *DNA Research*, 25(2):195–205, 2017.

[20] Fangwei Si, Dongyang Li, Sarah E. Cox, John T. Sauls, Omid Azizi, Cindy Sou, Amy B. Schwartz, Michael J. Erickstad, Yonggun Jun, Xintian Li, and Suckjoon Jun. Invariance of Initiation Mass and Predictability of Cell Size in Escherichia coli. *Current Biology*, 27(9):1278–1287, 2017.

[21] M Siwiak and P Zielenkiewicz. Transimulation - protein biosynthesis web service. *PLoS ONE*, 8(9):e73943, 2013.

[22] S. Sundararaj, A. Guo, habibi Nazhad B., Rouani M., Stothard P., Ellison M., and Wishart DS. The cybercell database (ccdb): a comprehensive, self-updating, relational database to coordinate and facilitate in silico modeling of escherichia coli. *Nucleic Acids Research*, 32(suppl1):D293–D295, 01 2004.

[23] Heykel Trabelsi, Mathilde Koch, and Jean-Loup Faulon. Building a minimal and generalizable model of transcription factorised biosensors: Showcasing flavonoids. *Biotechnology and Bioengineering*, 115:2292–2304, 2018.

[24] Pablo Ugalde-Salas, Elie Desmond-Le Quéméner, Jérôme Harmand, Alain Rapaport, and Théodore Bouchez. Insights from microbial transition state theory on monod's affinity constant. *Scientific Reports*, 10(1):5323, 2020.

[25] Andrea Y Weiße, Diego A Oyarzún, Vincent Danos, and Peter S Swain. Mechanistic links between cellular trade-offs, gene expression, and growth. *Proceedings of the National Academy of Sciences*, 112(9):E1038–E1047, 2015.

[26] Hong Zeng and Aidong Yang. Bridging substrate intake kinetics and bacterial growth phenotypes with flux balance analysis incorporating proteome allocation. *Scientific Reports*, 10(1):4283, 2020.

[27] Hai Zheng, Yang Bai, Meiling Jiang, Taku A. Tokuyasu, Xiongliang Huang, Fajun Zhong, Yuqian Wu, Xiongfei Fu, Nancy Kleckner, Terence Hwa, and Chenli Liu. General quantitative relations linking cell growth and the cell cycle in Escherichia coli. *Nature Microbiology*, 2020.
